# A rigorous theoretical and numerical analysis of a nonlinear reaction-diffusion epidemic model pertaining dynamics of COVID-19

**DOI:** 10.1038/s41598-024-56469-5

**Published:** 2024-04-04

**Authors:** Laiquan Wang, Arshad Alam Khan, Saif Ullah, Nadeem Haider, Salman A. AlQahtani, Abdul Baseer Saqib

**Affiliations:** 1 Department of Basic Courses, Changji Vocational and Technical College, Changji, 831100 China; 2 Department of Mathematics, University of Peshawar, Khyber Pakhtunkhwa, Pakistan; 3 Computer Engineering Department, College of Computer and Information Sciences, King Saud University, Riyadh, Saudi Arabia; 4 Faculty of Education, Department of Mathematics, Nangrahar University, Nangrahar, Afghanistan

**Keywords:** Spatial heterogeneity, Super-spreader events, Personal protection, Finite-difference operator-splitting approach, Threshold dynamics, Simulation, Computational biology and bioinformatics, Mathematics and computing, Computational biology and bioinformatics, Mathematics and computing

## Abstract

The spatial movement of the human population from one region to another and the existence of super-spreaders are the main factors that enhanced the disease incidence. Super-spreaders refer to the individuals having transmitting ability to multiple pathogens. In this article, an epidemic model with spatial and temporal effects is formulated to analyze the impact of some preventing measures of COVID-19. The model is developed using six nonlinear partial differential equations. The infectious individuals are sub-divided into symptomatic, asymptomatic and super-spreader classes. In this study, we focused on the rigorous qualitative analysis of the reaction-diffusion model. The fundamental mathematical properties of the proposed COVID-19 epidemic model such as boundedness, positivity, and invariant region of the problem solution are derived, which ensure the validity of the proposed model. The model equilibria and its stability analysis for both local and global cases have been presented. The normalized sensitivity analysis of the model is carried out in order to observe the crucial factors in the transmission of infection. Furthermore, an efficient numerical scheme is applied to solve the proposed model and detailed simulation are performed. Based on the graphical observation, diffusion in the context of confined public gatherings is observed to significantly inhibit the spread of infection when compared to the absence of diffusion. This is especially important in scenarios where super-spreaders may play a major role in transmission. The impact of some non-pharmaceutical interventions are illustrated graphically with and without diffusion. We believe that the present investigation will be beneficial in understanding the complex dynamics and control of COVID-19 under various non-pharmaceutical interventions.

## Introduction

COVID-19 caused by severe acute respiratory syndrome coronavirus was initially identified in China in 2019. It has been declared a pandemic by World Health Organization, as it spread very rapidly among the human population through different sources and reported million of confirmed cases accompanied by millions of deaths throughout the world^1^. The transmission of this infectious disease is difficult to control due to the uncertain nature of the virus. Many countries implement social distancing policies and avoid public gatherings, isolating the infected individuals to control the disease incidence. It is still a major threat to public health, although several vaccines are available now. The major factors that rapid the infection transmission are the super-spreaders and spatial movement of populations, since the disease may be transmitted faster in one place than another because of social contacts^2^. In earlier studies of disease epidemiology, it was assumed that susceptible hosts within a population had equal chances to become infected^3^. Further studies uncover the fact that heterogeneities in pathogen transmission with some individuals have a higher ability to infect others. The super-spreading events were found in many infectious diseases in history, such as tuberculosis, ebola, measles, HIV, hepatitis, and also found in the SARS pandemic^3^. Moreover, such events occur for a specific infectious disease when certain infected individuals produce more than the average number of secondary infected cases. Based on the analysis of the Centers for Disease Control and Prevention, an infected individual that produces more than 10 secondary infected cases is declared as a super-spreader^4^. Considering the above analysis, the super-spreaders have a significant role in infectious disease transmission.

The mathematical modeling approach is one of the important tools to analyze the impact of various aspects including super-spreading events on the dynamics of emerging and reemerging disease outbreaks. In this regard, various compartmental models have been presented and analyzed to uncover the epidemiological aspect of a disease^5^. Such as a fractional epidemic model in Caputo sense was formulated by Zafar et al.^6^ for understanding the dynamics of HIV/AIDS. The authors provide qualitative analysis of the proposed model and simulate it through a numerical scheme based on Newton’s polynomials. To analyze the dynamics of COVID-19 Baba et al.^7^ take into account fractional order epidemic model. Ibrahim et al.^8^ formulate an epidemic model to investigate the omicron variant of COVID-19 for real data from Thailand. The model is fractional based on Caputo derivative.

Mkhatshwa and Mummert in^4^ studied the impact of super-spreading events on the dynamics of the SARS infection. The reaction-diffusion epidemic models are considered helpful in analyzing the impact of spatio-temporal dynamics of infectious diseases outbreaks. Many researchers studied spatio-temporal modeling of COVID-19 to analyze the pandemic dynamics from different perspectives. For instance, Wang et al.^9^ investigate the reaction-diffusion epidemic model for spatio-temporal dynamics of infectious disease. Majid et al.^10^ presented a compartmental PDE model to investigate the spatial dynamics of the COVID-19 epidemic, whereas Zafar et al.^11^ present nonlinear fractional mathematical model to analyze tuberculosis using different fractional operators. A new reaction-diffusion problem is developed in^12^ to study the impact of infection transmission due to environmental load in a heterogeneous space. Fitzgibbon et al.^13^ presented a system of partial differential equations to investigate the dynamical study of the pandemic in a spatial inhomogeneous environment. Zheng et al.^14^ introduced a diffusive model to analyze the spatial spread of COVID-19 utilizing the incidence function of Beddington-DeAngelis type.

Kevrekidis et al.^15^ developed a new transmission epidemic model based on reaction-diffusion phenomena in order to explore the spatio-temporal transmission in two regions: the autonomous community of Andalusia in Spain and the mainland of Greece. To predict the long-time forecast of reaction-diffusion COVID-19 epidemic models, numerical treatment must be studied. Baba et al.^16^ proposed a fractional model to analyze COVID-19 with different variants. The authors proposed a fractional Adams-Bashforth scheme to obtain numerical solution.

Ahmed et al.^17^ analyze the SEIR reaction-diffusion model of infectious disease numerically by using the operator splitting non-standard finite-difference schemes. Many researchers utilize the operator splitting numerical schemes due to their positivity persevering property since the negative values of subpopulations in epidemic models are meaningless. The details are found in Refs.^18–21^. Most recently, a similar study has been carried out in^22^. In^22^, the authors introduced a reaction-diffusion epidemic model for the novel pandemic and explored the impact of various intervention measures in the presence of diffusion. Ahmed et al.^23^ investigate a well-known numerical approach, the fractional Euler method for approximate solution of fractional model based on Caputo derivative. Other similar literature can be found in^24,25^.

Motivated by the above literature, the present study develops a mathematical model that analyzes the role of super-spreaders on COVID-19 incidence and prevention with spatial and temporal impact. The present work is actually a spatial extension of the fractional order model^26^. To achieve our goals, initially a compartmental-based reaction-diffusion epidemic model is formulated. The qualitative analysis of the proposed model is carried out in detail and simulations are performed to figure out the influence of important parameters in the presence of diffusion. The article is organized the six main sections. In section “Description of the problem”, brief steps for the formulation of the model are presented. Basic qualitative analysis and stability of the model equilibria are discussed in section “Qualitative analysis of model”. In section “The model’s sensitivity analysis” sensitivity is explored. The numerical solution and detailed simulation of the model are discussed in section “Numerical treatment: solution and simulation”. Finally, section “Conclusion” accomplished the concluding remarks of the whole work.

## Description of the problem

This section briefly presents the procedure of model formulation. The assumptions taken in the problem construction are described. A reaction-diffusion mathematical model is presented to demonstrate the spatial and temporal dynamics of the disease. The present study is motivated by the fact that disease can spread more rapidly in certain regions compared to others, influenced by various factors such as public gatherings, weather conditions, social contacts, and so forth. The entire population is shown by $N(\tilde{t},\tilde{y})$ where $\tilde{t}\ge 0$ is any time instant and $\tilde{y}\in \Lambda = [a, b]$ with $a, b \in \mathbb {R}$ is spatial point. To construct the model, $N(\tilde{t},\tilde{y})$ is divided into six sub-groups shown by $S(\tilde{t},\tilde{y}), E(\tilde{t},\tilde{y}), I_1(\tilde{t},\tilde{y}), I_2(\tilde{t},\tilde{y}), I_3(\tilde{t},\tilde{y})$ and the recovered population $R(\tilde{t},\tilde{y})$. The description of each sub-group is described in Table 1. Thus, we have

$$\begin{aligned} N(t) = \int _{\Lambda }\bigg \{S(\tilde{t},\tilde{y})+E(\tilde{t},\tilde{y})+I_1(\tilde{t},\tilde{y})+I_2(\tilde{t},\tilde{y})+I_3(\tilde{t},\tilde{y})+R(\tilde{t},\tilde{y})\bigg \}d\tilde{y}. \end{aligned}$$

The transmissions among different classes are based on the following assumptions:

- Each newborn can get the infection. The susceptible class increased with newborns and reduced through infection and natural death.
- The susceptible get an infection after interacting with infected individuals and are moved to the exposed class which enhances it while reducing natural death and population completing their latency/incubation period.
- The fraction of the exposed population that has clinical symptoms is moved to the symptomatically infected class. This class is reduced with natural death, death due to infection, and recovery from infection.
- The fraction of the exposed population that has the ability to transfer multiple pathogens are super-spreaders and added to class super-spreaders. This class is also reduced with natural death, death due to infection, and recovery from infection.
- The fraction of the exposed population that has no clinical symptoms is moved to the asymptomatically infected class, which reduces with natural death and recovery from infection.
- The recovered class varies by moving individuals recovered from infection in any of the respective compartments and natural death.

Considering the above listed assumptions the spatio-temporal compartmental model describing the dynamics of COVID-19 is described as follows:

1 $$\begin{aligned} \left. \begin{array}{ll} \dfrac{\partial S}{\partial \tilde{t}} =D_{1}\dfrac{\partial ^2 S}{\partial \tilde{y}^2}+\Pi -\lambda S -\zeta S,\\ \\ \dfrac{\partial E}{\partial \tilde{t}} = D_{2}\dfrac{\partial ^2 E}{\partial \tilde{y}^2}+\lambda S -(r+\zeta )E,\\ \\ \dfrac{\partial I_1}{\partial \tilde{t}} = D_{3}\dfrac{\partial ^2 I_1}{\partial \tilde{y}^2}+r k_1 E -(\eta _1 +\zeta +\zeta _1)I_1,\\ \\ \dfrac{\partial I_2}{\partial \tilde{t}} = D_{4}\dfrac{\partial ^2 I_2}{\partial \tilde{y}^2}+r k_2 E -(\eta _2 +\zeta +\zeta _2)I_2,\\ \\ \dfrac{\partial I_3}{\partial \tilde{t}} = D_{5}\dfrac{\partial ^2 I_3}{\partial \tilde{y}^2}+r(1-k_1 -k_2) E-(\eta _3+\zeta )I_3,\\ \\ \dfrac{\partial R}{\partial \tilde{t}} =D_{6}\dfrac{\partial ^2 R}{\partial \tilde{y}^2}+\eta _3 I_3+\eta _2 I_2+\eta _1 I_1 -\zeta R, \end{array} \right\} \end{aligned}$$

where $$(\tilde{t},\tilde{y}) \in [0, T_{\text {max}}]\times [a, b]$$; $$T_{max}>0$$ and

$$\begin{aligned} \lambda (\tilde{t},\tilde{y}) = \beta \frac{I_1(\tilde{t},\tilde{y})+\psi I_3(\tilde{t},\tilde{y})}{N}+\beta _P\frac{ I_2(\tilde{t},\tilde{y})}{N}, \end{aligned}$$

denote the force of infection, which represents the transmission potential when susceptible individuals interact with infectious individuals $I_1, I_2$, and $I_3$. The detailed description of the parameters in model (1) is tabulated in Table 2, the coefficients of diffusivity are denoted by $D_{i}$ for $i = 1, 2,\ldots , 6$. Moreover, the following no-flux boundary conditions are considered for problem 1:

2 $$\begin{aligned} \left. \begin{array}{ll} &{}\xi _{\tilde{y}}(\tilde{t},-2) = 0,\\ &{}\xi _{\tilde{y}}(\tilde{t},2) = 0. \end{array} \right\} \end{aligned}$$

The symbol $\xi$ denotes the state variables of the model (1), where in (2), $\xi _{\tilde{y}}$ represent partial derivatives of each state variable of model (1) with respect to the spatial variable $\tilde{y}$.

### Initial conditions

To simulate model (1), the initial conditions (ICs) given by (3) and (4) are used. The ICs (4) are chosen based on^27^:

3 $$\begin{aligned}&\left. \begin{array}{rcl} \\ S(0, \tilde{y})&{}=&{}S_0 \ge 0, \\ \\ E(0, \tilde{y})&{}=&{}E_0 \ge 0, \\ \\ I_1(0, \tilde{y})&{}=&{}{I_1}_0 \ge 0, \\ \\ I_2(0, \tilde{y})&{}=&{}{I_2}_0 \ge 0, \\ \\ I_3(0, \tilde{y})&{}=&{}{I_3}_0 \ge 0, \\ \\ R(0, \tilde{y})&{}=&{}R_0\ge 0. \end{array}\right\} \end{aligned}$$

4 $$\begin{aligned}&\left. \begin{array}{rcl} S(0, \tilde{y})&{}=&{}S_0 \exp \left( -\left( \dfrac{\tilde{y}}{0.70}\right) ^2\right) , \\ E(0, \tilde{y})&{}=&{}E_0\exp \left( -\left( \dfrac{\tilde{y}}{0.30}\right) ^2\right) ,\\ I_1(0,x)&{}=&{}{I_1}_0\exp \left( -\left( \dfrac{\tilde{y}}{0.50}\right) ^2\right) ,\\ I_2(0, \tilde{y})&{}=&{}{I_2}_0\exp \left( -\left( \dfrac{\tilde{y}}{0.20}\right) ^2\right) ,\\ I_3(0, \tilde{y})&{}=&{}{I_3}_0,\\ R(0, \tilde{y})&{}=&{}R_0, \end{array}\right\} \end{aligned}$$

where $S_0 =34,806,871, E_0 = 20,000, {I_1}_0 = 3.0, {I_2}_0 = 110.0, {I_3}_0 = 0.0$ $R_0 = 0.0$, $\tilde{y}\in [a, b]$ and $a, b \in \mathbb {R}$ is the domain for the problem described in (1). The purpose of choosing the above set of ICs is to consider two types of population spatial distribution, i.e., homogeneous and heterogeneous environments to examine the role of diffusion in curtailing the infection in the aforementioned cases. The last two states, i.e., $I_{A0}$ and $R_0$ are assumed to be zero because an epidemic usually starts with a relatively small number of initially affected people. Frequently, by the time the pandemic is identified, these people might not have reached the asymptomatic or recovered stage.

**Table 1:** System (1) state variables.

State variables	Meaning
*S*	Susceptible population
*E*	Exposed population
$I_1$	Symptomatically Infected population
$I_2$	Super-spreaders
$I_3$	Asymptomatically Infected individuals
*R*	Individuals being Recovered from infection

**Table 2:** Biological details of the model parameters and respective numerical values.

Parameter	Description	Value/day
$\Pi$	Birth rate	*dN*(0)
$\zeta$	Natural mortality rate	$1/(74.87\times 365)$
$\psi$	Relative transmissibility due to $I_3$	0.100
$\beta$	Transmission rate	0.503
$\beta _P$	Transmission of infection of individuals in $I_2$	0.724
*r*	Incubation period	0.160
$k_1$	The exposed people entering $I_1$	0.472
$k_2$	The exposed people entering $I_2$	0.443
$\zeta _1$	Death rate in *I_1*(*t*) due to infection	0.012
$\zeta _2$	Death rate in $I_2$ due to infection	0.010
$\eta _1$	Rate of recovery $I_1$ class	0.327
$\eta _2$	Rate of removal/recovery in $I_2$ group	0.503
$\eta _3$	Rate of recovery $I_3$ class	0.060

The values are taken from^26^.

**Figure 1:**
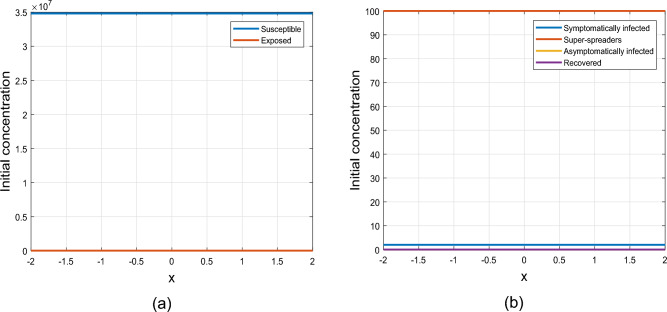
Graphical representation of uniform initial conditions (3).

**Figure 2:**
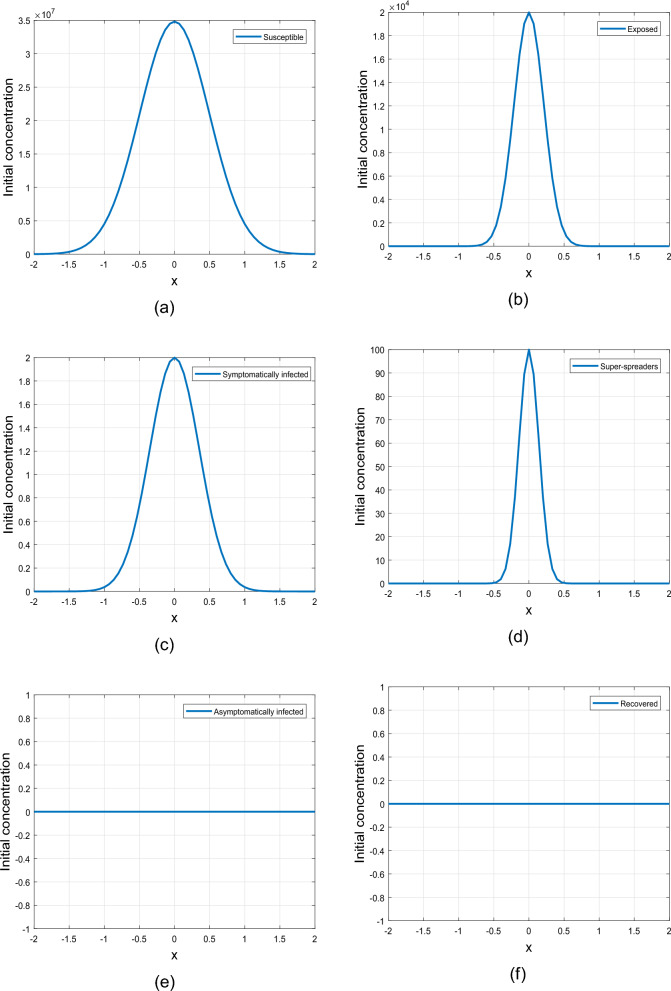
Graphical representation of uniform initial conditions (4).

The initial conditions profiles are presented in Figs. 1 and 2. The illustration in Fig. 1 depicts the ICs (3), showcasing a uniform distribution of population across the domain for each of the sub-populations under investigation in this study. The susceptible people class is considered to be larger than the rest of sub-classes, while the asymptomatic and recovered population are considered to be zero. Figure 2 indicates the ICs (4) with exposed, susceptible, symptomatic, and individuals in super-spreading class concentrated around the center of the interval $\left[ - 2, 2\right]$ and decreases exponentially towards the center (or origin) on both sides. The concentration of susceptible individuals at the origin significantly exceeds the concentrations of individuals in the exposed, symptomatic, and super-spreader classes. In addition, the concentrations of asymptomatic and recovered individuals are assumed to be zero.

## Qualitative analysis of model

This section presents qualitative analysis of the reaction-diffusion COVID-19 compartmental epidemic model (1). We proceed to prove the basic mathematical properties of the model solution as follows.

### Boundedness

One of the most important properties of an epidemic model is the solution boundedness. We take into consideration the approach described in^28^ in order to analyze the solution boundedness of the problem (1). The result is given in the following Theorem.

#### Theorem 3.1

The solution of the model (1) i.e., $(S(.,\tilde{t}), E(.,\tilde{t}), I_1(.,\tilde{t}), I_2(.,\tilde{t}), I_3(.,\tilde{t}), R(.,\tilde{t}))$ is bounded $\forall$ $\tilde{t}\ge 0$.

#### Proof

In order to prove the desired result, add all equations in the model (1)

$$\begin{aligned}&\dfrac{\partial }{\partial \tilde{t}}S(\tilde{t},\tilde{y})+\dfrac{\partial }{\partial \tilde{t}}E(\tilde{t},\tilde{y})+\dfrac{\partial }{\partial \tilde{t}}I_1(\tilde{t},\tilde{y})+\dfrac{\partial }{\partial \tilde{t}}I_2(\tilde{t},\tilde{y})+\dfrac{\partial }{\partial \tilde{t}}I_3(\tilde{t},\tilde{y})+\dfrac{\partial }{\partial \tilde{t}}R(\tilde{t},\tilde{y}), \\&= D_1\dfrac{\partial ^2}{\partial \tilde{y}^2}S(\tilde{t},\tilde{y})+D_2\dfrac{\partial ^2}{\partial \tilde{y}^2}E(\tilde{y}, t)+D_3\dfrac{\partial ^2}{\partial \tilde{y}^2}I_1(\tilde{t},\tilde{y})+D_4\dfrac{\partial ^2}{\partial \tilde{y}^2}I_2(\tilde{t},\tilde{y})+D_5\dfrac{\partial ^2}{\partial \tilde{y}^2}I_3(\tilde{t},\tilde{y})\\&\quad +D_6\dfrac{\partial ^2}{\partial \tilde{y}^2}R(\tilde{t},\tilde{y})+\Pi -\zeta (S(\tilde{t},\tilde{y})+E(\tilde{t},\tilde{y})+I_1(\tilde{t},\tilde{y})+I_2(\tilde{t},\tilde{y})+I_3(\tilde{t},\tilde{y})+R(\tilde{t},\tilde{y}))\\&\quad -\xi _{1}I_1(\tilde{t},\tilde{y})-\xi _{2}I_2(\tilde{t},\tilde{y}). \end{aligned}$$

Integrating over $\Lambda$, using the well known Divergence theorem^29^ and make use of no flux boundary conditions, which yield to

$$\begin{aligned}&\int _{\Lambda }\bigg \{\dfrac{\partial }{\partial \tilde{t}}S(\tilde{t},\tilde{y})+\dfrac{\partial }{\partial \tilde{t}}E(\tilde{t},\tilde{y})+\dfrac{\partial }{\partial \tilde{t}}I_1(\tilde{t},\tilde{y})+\dfrac{\partial }{\partial \tilde{t}}I_2(\tilde{t},\tilde{y})+\dfrac{\partial }{\partial \tilde{t}}I_3(\tilde{t},\tilde{y})+\dfrac{\partial }{\partial \tilde{t}}R(\tilde{t},\tilde{y}) \bigg \}d\tilde{y},\\&=\Pi |\Lambda |-d\int _{\Lambda }\bigg \{(S(\tilde{t},\tilde{y})+E(\tilde{t},\tilde{y})+I_1(\tilde{t},\tilde{y})+I_2(\tilde{t},\tilde{y})+I_3(\tilde{t},\tilde{y})+R(\tilde{t},\tilde{y}))\bigg \}d\tilde{y}\\&\quad -\int _{\Lambda }\bigg \{\xi _{1}I_1(\tilde{t},\tilde{y})+\xi _{2}I_2(\tilde{t},\tilde{y})\bigg \}d\tilde{y},\\&\le \Pi |\Lambda |-\zeta N(\tilde{t}).\\ \dfrac{d}{d\tilde{t}}N(\tilde{t})&=\Pi |\Lambda |-\zeta N(\tilde{t}). \end{aligned}$$

It gives

$$\begin{aligned}0\le N(\tilde{t})\le \dfrac{\Pi |\Lambda |}{\zeta }-\exp (-\zeta \tilde{t}) N(0), \quad \;\forall \; \ \tilde{t}\ge 0.\end{aligned}$$

Hence

$$\begin{aligned} \lim \limits _{t\rightarrow +\infty } N(t)\le \dfrac{\Pi |\Lambda |}{\zeta }. \end{aligned}$$

$$\square$$

### Invariant region

A positively invariant set for the system (1) is defined as follows:

$$\begin{aligned} \Phi = \bigg \{(S(\tilde{t},\tilde{y}), E(\tilde{t},\tilde{y}), I_1(\tilde{t},\tilde{y}), I_2(\tilde{t},\tilde{y}), I_3(\tilde{t},\tilde{y}), R(\tilde{t},\tilde{y})^{T}\in \mathbb {R}_+^6: N(\tilde{t})\le \dfrac{\Pi |\Lambda |}{d}\bigg \}\subset \mathbb {R}_+^6. \end{aligned}$$

### Model equilibria and the basic reproductive number

For derivation of the threshold parameter known as the basic reproductive number, we used the well-known approached considered in^30^. Model (1) possess two equilibria i.e. the disease-free equilibrium (DFE) and the endemic equilibrium (EE) represented by $\Xi _0$ and $\Gamma _{EE}$ respectively such that:

$$\begin{aligned} \Gamma _0 = (S_0, E_0, I_{1_0}, I_{2_0}, I_{3_0}, R_0)= \left( \Pi /\zeta , 0, 0, 0, 0, 0\right) . \end{aligned}$$

The EE is calculated as follows:

$$\begin{aligned} \Gamma _{EE} = (S^*, E^*, {I^*_{1}}, {I^*_{2}}, {I^*_{3}}, R^*), \end{aligned}$$

with the following analytical values

$$\begin{aligned} {\left\{ \begin{array}{ll} \\ S^{*} = \dfrac{\Pi \text{\c{N}}}{\text{\c{N}}\zeta +\mathfrak {R}^0-1}, \\ \\ E^{*} = \dfrac{\Pi (\mathfrak {R}^0-1)}{\alpha _1\left( \text{\c{N}}\zeta +\mathfrak {R}^0-1 \right) },\\ \\ I_1^{*} = \dfrac{\Pi \mathfrak {R}_{01}(\mathfrak {R}^0-1)}{\beta \left( \text{\c{N}}\zeta +\mathfrak {R}^0-1 \right) },\\ \\ I_2^{*} = \dfrac{\Pi \mathfrak {R}_{01}(\mathfrak {R}^0-1)}{\beta _P\left( \text{\c{N}}\zeta +\mathfrak {R}^0-1 \right) },\\ \\ I_3^{*} = \dfrac{\Pi \mathfrak {R}_{03}(\mathfrak {R}^0-1)}{\beta \psi \left( \text{\c{N}}\zeta +\mathfrak {R}^0-1\right) }, \\ \\ R^* = \dfrac{\Pi }{\zeta }\left[ \eta _1\dfrac{\mathfrak {R}_{01}}{\beta }+\eta _2\dfrac{\mathfrak {R}_{02}}{\beta _P}+\eta _3\dfrac{\mathfrak {R}_{03}}{\beta \psi } \right] \dfrac{(\mathfrak {R}^0-1)}{\left( \text{\c{N}}\zeta +\mathfrak {R}^0-1\right) }, \end{array}\right. } \end{aligned}$$

and

$$\begin{aligned} \text{\c{N}} = \frac{1}{r+\zeta }+\left( 1+\frac{\eta _1}{\zeta }\right) \frac{\mathfrak {R}_{01}}{\beta }+\left( 1+\frac{\eta _2}{\zeta }\right) \frac{\mathfrak {R}_{01}}{\beta _P}+\left( 1+\frac{\eta _3}{\zeta }\right) \frac{\mathfrak {R}_{03}}{\beta }. \end{aligned}$$

Moreover, the basic reproductive number $\mathfrak {R}^0$ is computed as follows:

The infectious classes in the proposed model (1) are $E, I_1, I_2$ and $I_3$, Henceforth, the vectors below present the transmission of newborn infections and the transitions between various classes.

$$\begin{aligned} {\textbf {F}}= \left( \begin{array}{c} \beta \frac{(I_1+\psi I_3)S}{N}+\beta _P\frac{ I_2 S}{N}\\ 0\\ 0\\ 0\\ \end{array}\right) , \quad \quad {\textbf {V}} = \left( \begin{array}{c} (r+\zeta )E\\ -r k_1 E+(\eta _1+\zeta +\zeta _{1})I_1\\ -r k_2 E+(\eta _2+\zeta +\zeta _{2})I_2\\ -r(1-k_1-k_2) E+(\eta _3+\zeta )I_3 \end{array} \right) , \end{aligned}$$

The Jacobian of above matrices are evaluated as:

$$\begin{aligned} \mathcal {F}= & {} \left( \begin{array}{cccc} 0 &{} \beta &{} \beta _\mathcal {P} &{} \psi \beta \\ 0 &{} 0 &{} 0 &{} 0\\ 0 &{} 0 &{} 0 &{} 0\\ 0 &{} 0 &{} 0 &{} 0\\ \end{array}\right) ,\\ \mathcal {V}= & {} \left( \begin{array}{cccc} r+\zeta &{} 0 &{} 0 &{} 0\\ -r k_1 &{} \eta _1+\zeta _1+\zeta &{} 0 &{} 0\\ -r k_1 &{} 0 &{} \eta _2+\zeta _2+\zeta &{} 0\\ -r(1-k_1-k_2) &{} 0 &{} 0 &{} \eta _3+\zeta \\ \end{array}\right) , \end{aligned}$$

and $N = \dfrac{\Pi }{\zeta }$ in case of disease-free equilibrium. Thus, the associated next generation matrix is,

$$\begin{aligned} \mathcal {F}\mathcal {V}^{-1} = \left( \begin{array}{cccc} \frac{r\beta \psi (1-k_1-k_2)}{(r+\zeta )(\eta _3+\zeta )}+\frac{r\beta k_1 }{(r+\zeta )(\eta _1+\zeta _{1}+\zeta )}+\frac{r\beta _P k_2 }{(r+\zeta )(\eta _2+\zeta _{2}+\zeta )} &{} \frac{\beta }{(\eta _1+\zeta _{1}+\zeta )} &{} \frac{\beta _P }{(\eta _2+\zeta _{2}+\zeta )} &{} \frac{\beta \psi }{(\eta _3+\zeta )}\\ 0 &{} 0 &{} 0 &{} 0\\ 0 &{} 0 &{} 0 &{} 0\\ 0 &{} 0 &{} 0 &{} 0 \end{array} \right) . \end{aligned}$$

The basic reproductive number which is the spectral radius of $FV^{-1}$ and is given by:

5 $$\begin{aligned} \mathfrak {R}^0 = \rho \left( FV^{-1}\right) = \mathfrak {R}_{01}+\mathfrak {R}_{02}+\mathfrak {R}_{03}, \end{aligned}$$

where

$$\begin{aligned} \mathfrak {R}_{01} =\frac{r\beta k_1 }{(r+\zeta )(\eta _1+\zeta +\zeta _{1})}, \mathfrak {R}_{02}= \frac{r\beta _P k_2 }{(r+\zeta )(\eta _2+\zeta +\zeta _{2})} \ \text {and} \ \mathfrak {R}_{03}= \frac{r\beta \psi (1-k_1-k_2)}{(r+\zeta )(\eta _3+\zeta )}. \end{aligned}$$

### Local stability

#### Theorem 3.2

The DFE $\Gamma _0$ is stable locally asymptomatically in $\Phi$ if $\mathfrak {R}^0<1$, otherwise it is unstable.

#### Proof

The Jacobian of (1) at the DFE point $\Gamma _0$ is

$$\begin{aligned} J(\Gamma _{0}) = \left( \begin{array}{cccccc} -\zeta &{} 0 &{} -\beta &{} -\beta _P &{} -\beta \psi &{} 0\\ 0 &{} -r-\zeta &{} \beta &{} \beta _P &{} \beta \psi &{} 0\\ 0 &{} r k_1 &{} -\eta _{1}-\zeta -\zeta _1 &{} 0 &{} 0 &{} 0 \\ 0 &{} r k_2 &{} 0 &{} -\eta _{2}-\zeta -\zeta _2 &{} 0 &{} 0 \\ 0 &{} r(1-k_{1}-k_{2}) &{} 0 &{} 0 &{} -\zeta -\eta _3 &{} 0 \\ 0 &{} 0 &{} \eta _1 &{} \eta _2 &{} \eta _3 &{} -\zeta \end{array}\right) , \end{aligned}$$

and the characteristic polynomial associated to above matrix is given as follows:

6 $$\begin{aligned} P(\varphi ) = (\zeta +\varphi )^2(\varphi ^{3}+c_{2}\varphi ^{2}+c_{1}\varphi +c_{0}), \end{aligned}$$

where,

$$\begin{aligned} c_{2}&= b_1l_2(1-\mathfrak {R}_{01})+l_1l_3(1-\mathfrak {R}_{02})+l_1l_4(1-\mathfrak {R}_{03})+l_2l_3+l_2l_4+l_3l_4,\\ c_{1}&= l_1l_2(l_3+l_4)(1-\mathfrak {R}_{01})+l_1l_3l_4(1-\mathfrak {R}_{03}-\mathfrak {R}_{02})+l_1l_4(l_3-l_2)\mathfrak {R}_{03}\\&\quad -l_1l_2l_3\mathfrak {R}_{02}, \\ c_{0}&=(r+\zeta )(\eta _3+\zeta )(\eta _1+\zeta _1+\zeta )(\eta _2+\zeta _2+\zeta ) \left( 1-\mathfrak {R}^0 \right) >0 \ \text {for} \ \mathfrak {R}^0<1, \end{aligned}$$

The values of $c_0, c_1, c_2$ in (6) are claimed to be positive under the condition $\mathfrak {R}^0<1$. Further, $c_1c_2-c_3>0$ confirming the Routh-Hurwitz conditions. Hence, the $\texttt {DFE}$ stable locally when $\mathfrak {R}^0<1$. $\square$

### Local Stability of endemic equilibria

To discuss the local stability of endemic equilibria $\Gamma _{EE}$, the model (1) is linearized at $\Gamma _{EE} = (S^*, E^*, I^*_{1}, I^*_{2}, I^*_{3}, R^* )$. For this purpose assume that

7 $$\begin{aligned} \left. \begin{array}{ll} \\ S(\tilde{t},\tilde{y}) &{}= \bar{S}(\tilde{t},\tilde{y})+S^*,\\ \\ E(\tilde{t},\tilde{y}) &{}= \bar{E}(\tilde{t},\tilde{y})+E^*,\\ \\ I_1(\tilde{t},\tilde{y}) &{}= \bar{I}_{1}(\tilde{t},\tilde{y})+I^*_{1},\\ \\ I_2(\tilde{t},\tilde{y}) &{}= \bar{I}_{2}(\tilde{t},\tilde{y})+I^*_{2},\\ \\ I_3(\tilde{t},\tilde{y}) &{}= \bar{I}_{3}(\tilde{t},\tilde{y})+I^*_{3},\\ \\ R(\tilde{t},\tilde{y}) &{}= \bar{R}(\tilde{t},\tilde{y})+R^*. \end{array} \right\} \end{aligned}$$

$\bar{S}(\tilde{t},\tilde{y}), \bar{E}(\tilde{t},\tilde{y}), \bar{I}_{1}(\tilde{t},\tilde{y}), \bar{I}_{2}(\tilde{t},\tilde{y}), \bar{I}_{3}(\tilde{t},\tilde{y})$ and $\bar{R}(\tilde{t},\tilde{y})$ are minimal perturbation. The linearized form of the problem (1) is given by (8),

8 $$\begin{aligned} \left. \begin{array}{ll} \\ \dfrac{\partial \bar{S}}{\partial \tilde{t}} &{}= D_1\dfrac{\partial ^2 \bar{S}}{\partial \tilde{y}^2}+g_{11}\bar{S}+g_{12}\bar{E}+g_{13}\bar{I}_{1}+g_{14}\bar{I}_2+g_{15}\bar{I}_3+g_{16}\bar{R},\\ \\ \dfrac{\partial \bar{E}}{\partial \tilde{t}} &{}= D_2\dfrac{\partial ^2 \bar{E}}{\partial \tilde{y}^2}+g_{21}\bar{S}+g_{22}\bar{E}+g_{23}\bar{I}_{1}+g_{24}\bar{I}_2+g_{25}\bar{I}_3+g_{26}\bar{R},\\ \\ \dfrac{\partial \bar{I}_1}{\partial \tilde{t}} &{}= D_3\dfrac{\partial ^2 \bar{I}_1}{\partial \tilde{y}^2}+g_{31}\bar{S}+g_{32}\bar{E}+g_{33}\bar{I}_{1}+g_{34}\bar{I}_2+g_{35}\bar{I}_3+g_{36}\bar{R},\\ \\ \dfrac{\partial \bar{I}_2}{\partial \tilde{t}} &{}= D_4\dfrac{\partial ^2 \bar{I}_2}{\partial \tilde{y}^2}+g_{41}\bar{S}+g_{42}\bar{E}+g_{43}\bar{I}_{1}+g_{44}\bar{I}_2+g_{45}\bar{I}_3+g_{46}\bar{R},\\ \\ \dfrac{\partial \bar{I}_3}{\partial \tilde{t}} &{}= D_5\dfrac{\partial ^2 \bar{I}_3}{\partial \tilde{y}^2}+g_{51}\bar{S}+g_{52}\bar{E}+g_{53}\bar{I}_{1}+g_{54}\bar{I}_2+g_{55}\bar{I}_3+g_{56}\bar{R},\\ \\ \dfrac{\partial \bar{R}}{\partial \tilde{t}} &{}= D_6\dfrac{\partial ^2 \bar{R}}{\partial \tilde{y}^2}+g_{61}\bar{S}+g_{62}\bar{E}+g_{63}\bar{I}_{1}+g_{64}\bar{I}_2+g_{65}\bar{I}_3+g_{66}\bar{R}, \end{array} \right\} \end{aligned}$$

such that

$$\begin{aligned} \begin{array}{ll} &{}g_{11} = -\dfrac{\beta }{N}\left( I^*_{1}+\psi I^*_{3}\right) -\dfrac{\beta _P}{N}I^*_{2}-\zeta , \ g_{12} = 0, \ g_{13} = -\dfrac{\beta }{N}S^*, \ g_{14} = -\dfrac{\beta _P}{N}S^*, \\ &{}g_{15} = -\dfrac{\beta \psi _{1}}{N}S^*,\hspace{0.3cm} g_{16} = 0,\hspace{0.3cm} g_{21} = \dfrac{\beta }{N}\left( I^*_{1}+\psi I^*_{3}\right) +\dfrac{\beta _P}{N}I^*_{2}, \hspace{0.3cm} g_{22} = -(r+\zeta ), \\ &{} g_{23} = \dfrac{\beta }{N}S^*, \hspace{0.3cm} g_{24} = \dfrac{\beta _P}{N}S^*, \hspace{0.3cm} g_{25} = \dfrac{\beta \psi }{N}S^*, \hspace{0.3cm} g_{26} = 0, \hspace{0.3cm} g_{31} = 0, \hspace{0.3cm} g_{32} = r k_1, \\ &{}g_{33} = -(\eta _{1}+\zeta +\zeta _1), \hspace{0.3cm} g_{34} = 0, \hspace{0.3cm} g_{35} = 0, \hspace{0.3cm} g_{36} = 0, \hspace{0.3cm} g_{41} = 0, \hspace{0.3cm} g_{42} = r k_2,\\ &{}g_{43} = 0, \hspace{0.3cm} g_{44} = -(\eta _{2}+\zeta +\zeta _2), \hspace{0.3cm} g_{45}=0, \hspace{0.3cm} g_{46}=0, \hspace{0.3cm} g_{51} = 0, \hspace{0.3cm} g_{56} = 0,\\ &{}g_{52} = r(1-k_1-k_2), \hspace{0.3cm} g_{53} = 0, \ g_{54} = 0, \ g_{55} = -(\eta _{3}+\zeta ), \hspace{0.3cm}g_{61} = 0,\\ &{}g_{62} = 0, \hspace{0.3cm} g_{55} = -(\eta _{3}+\zeta ), \hspace{0.3cm} g_{63} = \eta _1, \hspace{0.3cm} g_{64}=\eta _2, \hspace{0.3cm} g_{65}=\eta _3, \hspace{0.3cm} g_{66} = -\zeta . \end{array} \end{aligned}$$

Given that the linearized system (8) has a solution in Fourier series form, then

9 $$\begin{aligned} \left. \begin{array}{ll} \\ \bar{S}(\tilde{t},\tilde{y}) &{}= \sum \limits _{k}e^{\lambda t}b_{1k}\cos (k\tilde{y}), \\ \\ \bar{E}(\tilde{t},\tilde{y}) &{}= \sum \limits _{k}e^{\lambda t}b_{2k}\cos (k\tilde{y}), \\ \\ \bar{I}_{1}(\tilde{t},\tilde{y}) &{}= \sum \limits _{k}e^{\lambda t}b_{3k}\cos (k\tilde{y}), \\ \\ \bar{I}_{2}(\tilde{t},\tilde{y}) &{}= \sum \limits _{k}e^{\lambda t}b_{4k}\cos (k\tilde{y}), \\ \\ \bar{I}_{3}(\tilde{t},\tilde{y}) &{}= \sum \limits _{k}e^{\lambda t}b_{5k}\cos (k\tilde{y}), \\ \\ \bar{R}(\tilde{t},\tilde{y}) &{}= \sum \limits _{k}e^{\lambda t}b_{6k}\cos (k\tilde{y}). \end{array} \right\} \end{aligned}$$

In above, $k =\frac{n\pi }{2}$, with $n\in Z^{+}$, indicates wave-number for the node *n*. Using (9) in (8) yield to,

10 $$\begin{aligned} \left. \begin{array}{ll} \\ \sum \limits _{k}(g_{11}-k^2D_1-\lambda )b_{1k}+\sum \limits _{k}g_{12}b_{2k} +\sum \limits _{k}g_{13}b_{3k}+\sum \limits _{k}g_{14}b_{4k}+\sum \limits _{k}g_{15}b_{5k}+\sum \limits _{k}g_{16}b_{6k}&{}=0, \\ \\ \sum \limits _{k}g_{21}b_{1k}+\sum \limits _{k}(g_{22}-k^2D_{2}-\lambda )b_{2k}+\sum \limits _{k}g_{23}b_{3k}+\sum \limits _{k}g_{24}b_{4k}+\sum \limits _{k}g_{25}b_{5k}+\sum \limits _{k}g_{26}b_{6k}&{}=0, \\ \\ \sum \limits _{k}g_{31}b_{1k}+\sum \limits _{k}g_{32}b_{2k}+\sum \limits _{k}(g_{33}-k^2D_{I_1}-\lambda )b_{3k}+\sum \limits _{k}g_{34}b_{4k}+\sum \limits _{k}g_{35}b_{5k}+\sum \limits _{k}g_{36}b_{6k} &{}=0, \\ \\ \sum \limits _{k}g_{41}b_{1k}+\sum \limits _{k}g_{42}b_{2k}+\sum \limits _{k}g_{43}b_{3k}+\sum \limits _{k}(g_{44}-k^2D_4-\lambda )b_{4k}+\sum \limits _{k}g_{45}b_{5k}+\sum \limits _{k}g_{46}b_{6k} &{}=0, \\ \\ \sum \limits _{k}g_{51}b_{1k}+\sum \limits _{k}g_{52}b_{2k}+\sum \limits _{k}g_{53}b_{3k}+\sum \limits _{k}g_{54}b_{4k}+\sum \limits _{k}(g_{44}-k^2D_5-\lambda )b_{5k}+\sum \limits _{k}g_{56}b_{6k} &{}=0, \\ \\ \sum \limits _{k}g_{61}b_{1k}+\sum \limits _{k}g_{62}b_{2k}+\sum \limits _{k}g_{63}b_{3k}+\sum \limits _{k}g_{64}b_{4k}+\sum \limits _{k}g_{65}b_{5k}+\sum \limits _{k}(g_{66}-k^2D_{R}-\lambda )b_{6k} &{}=0. \end{array} \right\} \end{aligned}$$

The matrix *V* representing the variational matrix of (8) is

11 $$\begin{aligned} V = \left( \begin{array}{cccccc} -c_{11} &{} 0 &{} -g_{13}&{} -g_{14}&{} -g_{15}&{} 0\\ g_{21} &{} -c_{22}&{} g_{23} &{} g_{24}&{} g_{25}&{} 0\\ 0 &{}g_{23}&{}-c_{33}&{} 0&{} 0&{} 0\\ 0 &{}g_{42}&{}0&{}-c_{44}&{} 0&{} 0\\ 0 &{}g_{52}&{}0&{} 0&{}-c_{55}&{} 0\\ 0 &{}0&{}g_{63}&{} g_{64}&{} g_{65}&{}-c_{66}\\ \end{array}\right) , \end{aligned}$$

where,

$$\begin{aligned} c_{11}&= k^2D_1+g_{11}, \\ c_{22}&= k^2D_{2}+g_{22}, \\ c_{33}&= k^2D_3+g_{33}, \\ c_{44}&= k^2D_4+g_{44},\\ c_{55}&= k^2D_5+g_{55}, \\ c_{66}&= k^2D_{R}+g_{66}. \end{aligned}$$

The subsequent polynomial is,

12 $$\begin{aligned} P(\lambda ) = (\lambda +c_{66})(\lambda ^{5}+\mathcal {A}_{4}\lambda ^{4}+\mathcal {A}_{3}\lambda ^{3}+\mathcal {A}_{2}\lambda ^{2}+\mathcal {A}_{1}\lambda +\mathcal {A}_{0}). \end{aligned}$$

The relative coefficient values are;

$$\begin{aligned} \mathcal {A}_{4}&= c_{11}+k^{2}(D_2+D_3+D_4+D_5+D_6)+g_{11}+g_{22}+g_{33}+g_{44},\\ \mathcal {A}_{3}&= b_1+g_{22}\left( g_{33}\left( 1-\dfrac{\mathfrak {R}_{01}}{\mathfrak {R}^0}\right) +g_{44}\left( 1-\dfrac{\mathfrak {R}_{02}}{\mathfrak {R}^0}\right) +g_{55}\left( 1-\dfrac{\mathfrak {R}_{03}}{\mathfrak {R}^0}\right) \right) ,\\ \mathcal {A}_{2}&=b_2+b_3+c_{11}g_{22}\left( g_{33}\left( 1-\dfrac{\mathfrak {R}_{01}}{\mathfrak {R}^0}\right) +g_{44}\left( 1-\dfrac{\mathfrak {R}_{02}}{\mathfrak {R}^0}\right) +g_{55}\left( 1-\dfrac{\mathfrak {R}_{03}}{\mathfrak {R}^0}\right) \right) +g_{22}\\&\quad \times g_{33}(D_5+D_4)k^{2}\left( 1-\dfrac{\mathfrak {R}_{01}}{\mathfrak {R}^0}\right) +g_{22}g_{44}(D_5+D_3)k^{2}\left( 1-\dfrac{\mathfrak {R}_{02}}{\mathfrak {R}^0}\right) +g_{22}g_{55}k^{2}\\&\quad \times (D_3+D_4)\left( 1-\dfrac{\mathfrak {R}_{03}}{\mathfrak {R}^0}\right) +g_{22}g_{33}g_{44}\left( 1-\dfrac{\mathfrak {R}_{01}}{\mathfrak {R}^0}-\dfrac{\mathfrak {R}_{02}}{\mathfrak {R}^0} \right) +g_{22}g_{33}g_{55}\\&\quad \times \left( 1-\dfrac{\mathfrak {R}_{01}}{\mathfrak {R}^0}-\dfrac{\mathfrak {R}_{02}}{\mathfrak {R}^0} \right) +g_{22}g_{44}g_{55}\left( 1-\dfrac{\mathfrak {R}_{03}}{\mathfrak {R}^0}-\dfrac{\mathfrak {R}_{02}}{\mathfrak {R}^0}\right) +g_{21}g_{22}g_{33}\dfrac{\mathfrak {R}_{01}}{\mathfrak {R}^0}+ g_{21}g_{22}\\&\quad \times g_{55}\dfrac{\mathfrak {R}_{03}}{\mathfrak {R}^0}+g_{21}g_{22}g_{44}\dfrac{\mathfrak {R}_{02}}{\mathfrak {R}^0}, \\ \mathcal {A}_{1}&= b_4+g_{22}g_{33}D_5D_4k^{4}\left( 1-\dfrac{\mathfrak {R}_{01}}{\mathfrak {R}^0}\right) +c_{11}\left( g_{22}g_{33}(D_5+D_4)k^{2}\left( 1-\dfrac{\mathfrak {R}_{01}}{\mathfrak {R}^0}\right) \right) \\&\quad +g_{22}g_{44}D_5D_3k^{4}\left( 1-\dfrac{\mathfrak {R}_{02}}{\mathfrak {R}^0}\right) +c_{11}\left( g_{22}g_{44}(D_5+D_3)k^{2}\left( 1-\dfrac{\mathfrak {R}_{02}}{\mathfrak {R}^0}\right) \right) +c_{11}\\&\quad \times \left( g_{22}g_{55}(D_4+D_3)k^{2}\left( 1-\dfrac{\mathfrak {R}_{03}}{\mathfrak {R}^0}\right) \right) +c_{11}g_{22}g_{33}g_{44}\left( 1-\dfrac{\mathfrak {R}_{01}}{\mathfrak {R}^0}-\dfrac{\mathfrak {R}_{02}}{\mathfrak {R}^0}\right) +c_{11} \\&\quad \times g_{22}g_{55}\left( g_{33}\left( 1-\dfrac{\mathfrak {R}_{03}}{\mathfrak {R}^0}-\dfrac{\mathfrak {R}_{01}}{\mathfrak {R}^0}\right) +g_{44}\left( 1-\dfrac{\mathfrak {R}_{03}}{\mathfrak {R}^0}-\dfrac{\mathfrak {R}_{02}}{\mathfrak {R}^0}\right) \right) +g_{22}g_{33}g_{44}D_5k^{2}\\&\quad \times \left( 1-\dfrac{\mathfrak {R}_{01}}{\mathfrak {R}^0}-\dfrac{\mathfrak {R}_{02}}{\mathfrak {R}^0}\right) +g_{22}g_{55}D_4D_3k^{4}\left( 1-\dfrac{\mathfrak {R}_{03}}{\mathfrak {R}^0}\right) +g_{22}g_{33}g_{55}D_4k^{2}\\&\quad \times \left( 1-\dfrac{\mathfrak {R}_{03}}{\mathfrak {R}^0}-\dfrac{\mathfrak {R}_{01}}{\mathfrak {R}^0}\right) +g_{22}g_{44}g_{55}D_3k^{2}\left( 1-\dfrac{\mathfrak {R}_{03}}{\mathfrak {R}^0}-\dfrac{\mathfrak {R}_{02}}{\mathfrak {R}^0}\right) +g_{21}(c_{44}+c_{55})g_{22}\\&\quad \times g_{33}\dfrac{\mathfrak {R}_{01}}{\mathfrak {R}^0}+g_{21}(c_{33}+c_{44})g_{22}g_{55}\dfrac{\mathfrak {R}_{03}}{\mathfrak {R}^0}+g_{21}(c_{33}+c_{55})g_{22}g_{44}\dfrac{\mathfrak {R}_{02}}{\mathfrak {R}^0},\\ \mathcal {A}_{0}&= b_5 +c_{11}g_{22}g_{44}D_5D_3k^{4}\left( 1-\dfrac{\mathfrak {R}_{01}}{\mathfrak {R}^0}\right) +c_{11}g_{22} g_{55}D_4D_3k^{4}\left( 1-\dfrac{\mathfrak {R}_{03}}{\mathfrak {R}^0}\right) +c_{11}g_{22}\\&\quad \times g_{33}g_{44}D_5k^{2}\left( 1-\dfrac{\mathfrak {R}_{02}}{\mathfrak {R}^0}\right) +c_{11}g_{22}g_{33}g_{55}D_4k^{2}\left( 1-\dfrac{\mathfrak {R}_{03}}{\mathfrak {R}^0}\right) +c_{11}g_{22}g_{44}g_{55}D_3k^{2}\\&\quad \times \left( 1-\dfrac{\mathfrak {R}_{03}}{\mathfrak {R}^0}\right) +c_{11}g_{22}g_{33}g_{44}g_{55}\left( 1-\dfrac{\mathfrak {R}_{03}}{\mathfrak {R}^0}-\dfrac{\mathfrak {R}_{02}}{\mathfrak {R}^0}\right) -c_{11}g_{22}g_{33}D_5D_4k^{4}\dfrac{\mathfrak {R}_{01}}{\mathfrak {R}^0}\\&\quad -c_{11}g_{22}g_{33}g_{44}k^{2}\left( D_4\dfrac{\mathfrak {R}_{01}}{\mathfrak {R}^0}+D_3\dfrac{\mathfrak {R}_{02}}{\mathfrak {R}^0}\right) -c_{11}c_{55}g_{22}g_{44}^{2}\dfrac{\mathfrak {R}_{01}}{\mathfrak {R}^0}. \end{aligned}$$

The values of $b_i$, where $i=1,\ldots ,5$ are given below:

$$\begin{aligned} b_1&= c_{11} (c_{22}+c_{33}+c_{44}+c_{55})+g_{33}\left( D_2k^{2}+c_{44}+c_{55}\right) +g_{44}k^{2}\left( D_2+D_3+D_5\right) \\&\quad +g_{55}\left( (D_2+D_3)k^{2}+c_{44}\right) +k^{4}\left( D_5(D_4+D_{I_E})+(D_5+D_4)(D_3+D_{I_E})\right) \\&\quad +g_{22}k^{2}\left( D_3+D_4+D_5\right) ,\\ b_2&=c_{11}\left( k^{4}\left( D_5(D_4+D_{I_E})+(D_5+D_4)(D_3+D_{I_E})\right) +g_{44}k^{2}\left( D_2+D_5+D_3\right) \right) \\&\quad +c_{11}g_{22}k^{2}\left( D_3+D_4+D_5\right) ,\\ b_3&=c_{11}\left( g_{44}((D_2+D_3)k^{2}+c_{44})+g_{33}(D_2k^{2}+c_{44}+c_{55})\right) +(D_4D_5(D_2+D_3))k^{6}\\&\quad +(D_2D_3(D_5+D_4))k^{6}+g_{22}(D_4D_4+D_5(D_4+D_3))k^{2}+g_{33}g_{44}k^{2}(D_2+D_5)\\&\quad +g_{33}(D_5D_4+D_{I_E}(D_5+D_4))k^{2}+g_{44}k^{4}(D_5D_3+D_{I_E}(D_5+D_3))\\&\quad +g_{55}k^{4}(D_2D_4+D_3(D_2+D_4))+g_{44}g_{55}k^{2}(D_2+D_3)+g_{33}g_{55}(D_2k^{2}+c_{44}),\\ b_4&=c_{11}\left( D_5D_4(D_2+D_3)+D_2D_3(D_5+D_4)k^{6}+\left( (g_{22}+g_{55})D_4D_3\right) k^{4} \right) \\&\quad +c_{11}\left( (D_4+D_3)(g_{22}D_5+g_{55}D_2)k^{4}+g_{44}\left( D_5D_3+D_{I_E}(D_5+D_3)\right) k^{4} \right) \\&\quad +c_{11}\left( g_{44}\left( D_5D_4+D_{I_E}(D_5+D_4)\right) k^{4}+g_{55}(g_{33}(D_2+D_4)+g_{44}(D_2+D_5))k^{2} \right) \\&\quad +c_{11}\left( g_{44}g_{55}+2(D_2+D_3)k^{2}\right) +g_{22}D_3D_5D_4k^{6}+c_{33}c_{44}c_{55}D_2k^{2},\\ b_5&=g_{21}c_{22}c_{33}c_{44}c_{55}+c_{11}\left( c_{33}k^{2}(g_{22}D_5D_5k^{2})+D_2(c_{44}+c_{55}) \right) . \end{aligned}$$

The coefficients $\mathcal {A}_{i}$, $i = 0,\ldots , 4$, of *P* given by (13) are positive, if $\mathfrak {R}^0>1$. Further, it also satisfies the Routh-Hurwitz stability conditions for a polynomial having degree five, i.e.,

$$\begin{aligned}&\mathcal {A}_{4}\mathcal {A}_{3}\mathcal {A}_{2}-\mathcal {A}_{2}^{2}-\mathcal {A}_{4}^{2}\mathcal {A}_{1}>0,\\&\left( \mathcal {A}_{4}\mathcal {A}_{1}-\mathcal {A}_{0}\right) \left( \mathcal {A}_{4}\mathcal {A}_{3}\mathcal {A}_{2}-\mathcal {A}_{2}^2-\mathcal {A}_{4}^2\mathcal {A}_{1}\right) -\mathcal {A}_{0}(\mathcal {A}_{4}\mathcal {A}_{3}\mathcal {A}_{2})^2-\mathcal {A}_{4}\mathcal {A}_{0}^2>0. \end{aligned}$$

Therefore, it is asserted that $\Gamma _{EE}$ is stable locally for $\mathfrak {R}^{0}>1$.

### Global stability of the model

In the subsequent section, we will investigate the stability of the problem (1) in global case at the steady state $\Gamma _{0}$ using a nonlinear Lyapunov stability approach.

#### Theorem 3.3

If $\mathfrak {R}^0<1$ then the DFE $\Gamma _{0}$ of the system (1) is globally stable in $\Phi$.

#### Proof

The Lyapunov-type function is define as

$$\begin{aligned} V(\tilde{t}) = \int _{\Lambda }\biggl \{E(\tilde{t},\tilde{y})+j_1I_1(\tilde{t},\tilde{y})+j_2I_2(\tilde{t},\tilde{y})+j_3I_3(\tilde{t},\tilde{y})\biggr \}d\tilde{y}, \end{aligned}$$

with

$$\begin{aligned} j_1 = \dfrac{\beta }{\eta _{1}+\zeta +\zeta _1}, \ j_2 = \dfrac{\beta }{\eta _{2}+\zeta +\zeta _2}, \ \hbox {and} \ j_3 = \dfrac{\psi \beta }{\eta _{1}+\zeta }. \end{aligned}$$

Differentiating $V(\tilde{t},\tilde{y})$ with the solution of (1) as follows:

$$\begin{aligned} \dfrac{dV}{d\tilde{t}}&= \int _{\Lambda }\biggl \{D_{2}\dfrac{\partial ^2 E(\tilde{t},\tilde{y})}{\partial \tilde{y}^2}+j_1D_{3}\dfrac{\partial ^2 I_1(\tilde{t},\tilde{y})}{\partial \tilde{y}^2}+j_2D_{4}\dfrac{\partial ^2 I_2(\tilde{t},\tilde{y})}{\partial \tilde{y}^2}+j_3D_{5}\dfrac{\partial ^2 I_2(\tilde{t},\tilde{y})}{\partial \tilde{y}^2}\biggr \}dx\\&\quad +\int _{\Lambda }\biggl \{j_1r k_1+j_2r k_2+j_3r(1-k_1-k_2)-(r+\zeta ) \biggr \}E(\tilde{t},\tilde{y})d\tilde{y}\\&\quad +\int _{\Lambda }\biggl \{\beta \frac{I_1(\tilde{t},\tilde{y})S(\tilde{t},\tilde{y})}{N(\tilde{t},\tilde{y})}+\beta \psi \frac{I_3(\tilde{t},\tilde{y})S(\tilde{t},\tilde{y})}{N(\tilde{t},\tilde{y})}+\beta _P\frac{I_2(\tilde{t},\tilde{y})S(\tilde{t},\tilde{y})}{N(\tilde{t},\tilde{y})} \biggr \}d\tilde{y}\\&\quad -\int _{\Lambda }\biggl \{j_1(\eta _{1}+\zeta +\zeta _{1})I_1+j_2(\eta _{2}+\zeta +\zeta _{2})I_2+j_3(\eta _{3}+\zeta )I_3\biggr \}d\tilde{y}. \end{aligned}$$

As, $S(\tilde{t},\tilde{y})\le N(\tilde{t},\tilde{y})$ for $\tilde{y}\in \Lambda$ and $\tilde{t}\ge 0$, therefore,

$$\begin{aligned} \dfrac{dV}{d\tilde{t}}&\le \int _{\Lambda }\biggl \{D_{2}\dfrac{\partial ^2 E(\tilde{t},\tilde{y})}{\partial \tilde{y}^2}+D_{3}\dfrac{\partial ^2 I_1(\tilde{t},\tilde{y})}{\partial \tilde{y}^2}+D_{4}\dfrac{\partial ^2 I_2(\tilde{t},\tilde{y})}{\partial \tilde{y}^2}+D_{5}\dfrac{\partial ^2 I_2(\tilde{t},\tilde{y})}{\partial \tilde{y}^2}\biggr \}d\tilde{y}\\&\quad +\int _{\Lambda }\biggl \{j_1r k_1+j_2r k_2+j_3r(1-k_1-k_2)-(r+\zeta ) \biggr \}E(\tilde{t},\tilde{y})d\tilde{y}\\&\quad +\int _{\Lambda }\biggl \{\beta I_1(\tilde{t},\tilde{y})+\beta \psi I_3(\tilde{t},\tilde{y})+\beta _PI_2(\tilde{t},\tilde{y}) \biggr \}d\tilde{y}\\&\quad -\int _{\Lambda }\biggl \{j_1(\eta _{1}+\zeta +\zeta _{1})I_1+j_2(\eta _{2}+\zeta +\zeta _{2})I_2+j_3(\eta _{3}+\zeta )I_3\biggr \}d\tilde{y}, \\ \dfrac{dV}{d\tilde{t}}&\le \int _{\Lambda }\biggl \{D_{2}\dfrac{\partial ^2 E(\tilde{t},\tilde{y})}{\partial \tilde{y}^2}+D_{3}\dfrac{\partial ^2 I_1(\tilde{t},\tilde{y})}{\partial \tilde{y}^2}+D_{4}\dfrac{\partial ^2 I_2(\tilde{t},\tilde{y})}{\partial \tilde{y}^2}+D_{5}\dfrac{\partial ^2 I_2(\tilde{t},\tilde{y})}{\partial \tilde{y}^2}\biggr \}d\tilde{y}\\&\quad +\int _{\Lambda }\biggl \{j_1r k_1+j_2r k_2+j_3r(1-k_1-k_2)-(r+\zeta ) \biggr \}E(\tilde{t},\tilde{y})d\tilde{y}\\&\quad +\int _{\Lambda }\biggl \{(\beta -j_1(\eta _{1}+\zeta +\zeta _{1}))I_1(\tilde{t},\tilde{y})+(\beta \psi -j_2(\eta _{2}+\zeta +\zeta _{2}))I_3(\tilde{t},\tilde{y})\biggr \}d\tilde{y}\\&\quad +\int _{\Lambda }\biggl \{(\beta _P-j_3(\eta _{3}+\zeta ))I_2(\tilde{t},\tilde{y})\biggr \}d\tilde{y}. \end{aligned}$$

Using, the criterions described in (2), we have

$$\begin{aligned} \dfrac{dV}{d\tilde{t} }&\le (r+\zeta )\int _{\Lambda }\biggl \{j_1\dfrac{r k_1}{(r+\zeta )}+j_2\dfrac{r k_2}{(r+\zeta )}+j_3\dfrac{r(1-k_1-k_2)}{(r+\zeta )}-1 \biggr \}E(\tilde{t},\tilde{y})d\tilde{y}\\&\le (r+\zeta )(\mathfrak {R}^0-1)\int _{\Lambda }E(\tilde{t},\tilde{y})d\tilde{y}. \end{aligned}$$

It is obvious $\dfrac{dV}{d\tilde{t} }<0$ $\forall$ $\tilde{t} \ge 0$ and $\tilde{y}\in \Lambda$ $\Leftrightarrow$ $\mathfrak {R}^0<1$. Further, $\dfrac{dV}{d\tilde{t} }=0$ if and only if $E(\tilde{t},\tilde{y})\rightarrow 0$, then the model (1) implies that $I_1(\tilde{t},\tilde{y})\rightarrow 0$, $I_2(\tilde{t},\tilde{y})\rightarrow 0$, $I_2(\tilde{t},\tilde{y})\rightarrow 0$, $R( t,\tilde{y})\rightarrow 0$ and $S(\tilde{t},\tilde{y})\rightarrow \dfrac{\Pi }{\zeta}$ $(S, E, I_1, I_2, I_3, R) \rightarrow \left( \dfrac{\Pi }{\zeta }, 0, 0, 0, 0, 0\right)$. In a result we concluded that the largest compact invariant set within the region $\left\{ (S, E, I_1, I_2, I_3, R): \dfrac{dV}{d\tilde{t}}=0 \right\}$ is $\Gamma _0$. By employing the well-established LaSalle’s principle, $\Gamma _0$ is globally asymptotically stable under the condition $\mathfrak {R}^0<1$. $\square$

## The model’s sensitivity analysis

Sensitivity analysis of epidemiological systems plays a crucial role in understanding the impact of system embedded parameters on disease incidence and prevalence. It helps to elucidate the significance of various parameters in shaping the dynamics of the epidemic. Due to the potential for errors in the collocation of data and uncertainties in parameter values, such analysis becomes essential for assessing the robustness of the respective model predictions. The sensitivity analysis provides insights into the reliability and stability of the model under different scenarios. To identify certain parameters that play a critical role and exert a significant impact on $\mathfrak {R}^0$, computing their sensitivity indices proves to be effective. These parameters become prime targets for intervention strategies aimed at controlling the epidemic. The sensitivity index of $\mathfrak {R}^0$ relative t to the system parameter $\Theta$ is defined as:

13 $$\begin{aligned} \Psi ^{\mathfrak {R}^0}_\Theta = \frac{\Theta }{\mathfrak {R}^0}\frac{\partial \mathfrak {R}^0}{\partial \Theta }, \end{aligned}$$

where $\Theta$ shows the system parameter involved in (1). Sensitivity indices of $\mathfrak {R}^0$ analytically represents as:

$$\begin{aligned} \Psi ^{\mathfrak {R}^0}_\beta&= \frac{1}{\mathfrak {R}^0}[\mathfrak {R}^0-\mathfrak {R}_{02}]>0,\\ \Psi ^{\mathfrak {R}^0}_r&= \frac{\zeta }{r+\zeta }>0,\\ \Psi ^{\mathfrak {R}^0}_{\beta _P}&= \frac{\mathfrak {R}_{02}}{\mathfrak {R}^0}>0,\\ \Psi ^{\mathfrak {R}^0}_{\zeta }&= -d\left[ 1+\left( \frac{r+\zeta }{\eta _{1}+\zeta +\zeta _{1}}\right) \frac{\mathfrak {R}_{01}}{\mathfrak {R}^0}+\left( \frac{r+\zeta }{\eta _{2}+\zeta +\zeta _{2}}\right) \frac{\mathfrak {R}_{02}}{\mathfrak {R}^0}+\left( \frac{r+\zeta }{\eta _{3}+\zeta }\right) \frac{\mathfrak {R}_{03}}{\mathfrak {R}^0}\right]<0,\\ \Psi ^{\mathfrak {R}^0}_{k_{1}}&= \frac{1}{\mathfrak {R}^0}\left[ \mathfrak {R}_{01}-\frac{r\beta \psi k_{1}}{(\eta _{3}+\zeta )(r+\zeta )} \right]>0,\\ \Psi ^{\mathfrak {R}^0}_{k_{2}}&= \frac{1}{\mathfrak {R}^0}\left[ \mathfrak {R}_{02}-\frac{r\beta \psi k_{2}}{(\eta _{3}+\zeta )(r+\zeta )} \right]>0,\\ \Psi ^{\mathfrak {R}^0}_{\zeta _{1}}&= -\frac{\zeta _{1}}{(\eta _{1}+\zeta +\zeta _{1})}\left[ \frac{\mathfrak {R}_{01}}{\mathfrak {R}^0}\right]<0,\\ \Psi ^{\mathfrak {R}^0}_{\zeta _{2}}&= -\frac{\zeta _{2}}{(\eta _{2}+\zeta +\zeta _{2})}\left[ \frac{\mathfrak {R}_{02}}{\mathfrak {R}^0}\right]<0,\\ \Psi ^{\mathfrak {R}^0}_{\eta _{1}}&= -\frac{\eta _{1}}{(\eta _{1}+\zeta +\zeta _{1})}\left[ \frac{\mathfrak {R}_{01}}{\mathfrak {R}^0}\right]<0,\\ \Psi ^{\mathfrak {R}^0}_{\eta _{2}}&= -\frac{\eta _{2}}{(\eta _{2}+\zeta +\zeta _{2})}\left[ \frac{\mathfrak {R}_{02}}{\mathfrak {R}^0}\right]<0,\\ \Psi ^{\mathfrak {R}^0}_{\eta _{3}}&= -\frac{\eta _{3}}{(\eta _{3}+\zeta )}\left[ \frac{\mathfrak {R}_{03}}{\mathfrak {R}^0}\right] <0,\\ \Psi ^{\mathfrak {R}^0}_{\psi }&= \frac{\mathfrak {R}_{03}}{\mathfrak {R}^0}>0. \end{aligned}$$

Furthermore, to fulfill these analytical results, the sensitivity indices are evaluated numerically utilizing parameter values given in Table 1, and numerical indices are provided in Table 3. The above analysis indicates that the parameters $\beta , r, \beta _{P}, k_{1}, k_{2}$, and $\psi$ exhibit positive sensitivity indices, indicating their role in enhancing $\mathfrak {R}^0$ for larger values. Conversely, parameters $\zeta , \zeta _{1}, \zeta _{2}, \eta _{1}, \eta _{2}$, and $\eta _{3}$ display negative indices, suggesting an inverse relationship with $\mathfrak {R}^0$. Thus, an increase in these parameter values would decrease $\mathfrak {R}^0$. Given their larger indices, these parameters, $\beta$ and $\beta {P}$ are the most sensitive. Small increments in these parameter’s values could significantly elevate the value of $\mathfrak {R}^0$. In biological context, the average number of contacts per person within a certain time interval is represented by the contact rate $\beta$. Elevated $\beta$ values signify an increased probability of transmission during physical interaction. On the other hand, the high sensitivity of the parameter represents the transmission due super-spreader suggesting that the contribution of individuals with a high capacity for transmission, has a significant influence on the overall dynamics of the epidemic.

**Table 3:** Sensitivity indices of model parameters versus $\mathfrak {R}^0$.

Parameters	Index value
$\beta$	+ 0.5523
*r*	+ 0.0003
$\beta _{P}$	+ 0.4476
$\zeta$	− 0.0004
$k_1$	+ 0.2184
$k_2$	+ 0.1821
$\zeta _{1}$	− 0.1833
$\zeta _{2}$	− 0.0087
$\eta _{1}$	− 0.4828
$\eta _{2}$	− 0.4389
$\eta _{3}$	− 0.0510
$\psi$	+ 0.05106

## Numerical treatment: solution and simulation

The present part of the manuscript investigates the iterative solution of the system (1) using finite difference operator-splitting (FDOS) approximation technique presented in^17,18^. According to the Von Neumann stability criteria, the spatial and time step size for this purpose are considered as $\Pi \tilde{y} = 0.06$, and $\Pi \tilde{t} = 0.02$ days. The diffusivity coefficients have been taken as $D_{1}=0.000050, D_{2}=0.00050, D_{3}=0.00010, D_{4}=0.0010, D_{5} = 0.00010, D_{6}=0$. The proposed scheme steps are presented as fallow:

### Solution scheme

The operator splitting iterative scheme is one of the efficient numerical techniques in the field of numerical analysis. This method is successfully applied for the solution of nonlinear partial differential equations in order to handle the complexity and non-linearity. This scheme is generally based on the splitting approach of differential operators into sub-operators. It results in the splitting of of problem under consideration into sub-problems corresponding to a particular physical phenomenon. In this investigation, the mentioned scheme is applied to solve the reaction-diffusion compartmental epidemic model for the dynamics of COVID-19 described in (1). Different population groups interact with one another and diffuse spatially in uni-direction requiring different temporal steps. Therefore, considering the operator-splitting approach in^17,18^, splitting the time dependent operator is useful which shift model (1) in to two sub-systems. The nonlinear reaction problem utilized for the time-step $t_0$ to $\frac{1}{2}dt$ is

14 $$\begin{aligned} \left. \begin{array}{ll} \\ \dfrac{1}{2}\dfrac{\partial S}{\partial \tilde{t}} &{}=\Pi -\lambda S -d S, \\ \\ \dfrac{1}{2}\dfrac{\partial E}{\partial \tilde{t}} &{}= \lambda S -l_1E,\\ \\ \dfrac{1}{2}\dfrac{\partial I_1}{\partial \tilde{t}} &{}= r k_1 E -l_{2}I_1,\\ \\ \dfrac{1}{2}\dfrac{\partial I_2}{\partial \tilde{t}} &{}= r k_2 E -l_3I_2,\\ \\ \dfrac{1}{2}\dfrac{\partial I_3}{\partial \tilde{t}} &{}= r(1-k_1 -k_2) E-l_4I_3,\\ \\ \dfrac{1}{2}\dfrac{\partial R}{\partial \tilde{t}} &{}= \eta _3 I_3+\eta _2 I_2+\eta _1 I_1 -\zeta R. \end{array} \right\} \end{aligned}$$

Moreover, the diffusion problem in linear case utilized for time-step $\frac{1}{2}dt$ to $t^{n}$ is as follows

15 $$\begin{aligned} \left. \begin{array}{ll} \\ \dfrac{1}{2}\dfrac{\partial S}{\partial \tilde{t}} &{}= \dfrac{\partial ^2 S}{\partial \tilde{y}^2}D_{1},\\ \\ \dfrac{1}{2}\dfrac{\partial E}{\partial \tilde{t}} &{}= \dfrac{\partial ^2 E}{\partial \tilde{y}^2}D_{2},\\ \\ \dfrac{1}{2}\dfrac{\partial I_1}{\partial \tilde{t}} &{}= \dfrac{\partial ^2 I_1}{\partial \tilde{y}^2}D_{3},\\ \\ \dfrac{1}{2}\dfrac{\partial I_2}{\partial \tilde{t}} &{}= \dfrac{\partial ^2 I_2}{\partial \tilde{y}^2}D_{4},\\ \\ \dfrac{1}{2}\dfrac{\partial I_3}{\partial \tilde{t}} &{}= \dfrac{\partial ^2 I_3}{\partial \tilde{y}^2}D_{5},\\ \\ \dfrac{1}{2}\dfrac{\partial R}{\partial \tilde{t}} &{}= \dfrac{\partial ^2 R}{\partial \tilde{y}^2}D_{6}.\\ \end{array} \right\} \hspace{2.80cm} \end{aligned}$$

Now make use of finite-difference approximations, the time derivative with first order in (14) and (15) is approximated by as follows

16 $$\begin{aligned} \dfrac{\partial \xi ^{n}_{j}}{\partial \tilde{t}} = \dfrac{\xi ^{n+1}_{j}-\xi ^{n}_{j}}{d\tilde{t}}, \end{aligned}$$

while the spatial derivative with second-order in the above system (15) is approximated by second-order central finite-difference described as follows:

17 $$\begin{aligned} \dfrac{\partial ^2 \xi ^{n}_{j}}{\partial \tilde{y}^2} = \dfrac{\xi ^{n}_{j-1}-\xi ^{n}_{j}+\xi ^{n}_{j+1}}{d\tilde{y}}. \end{aligned}$$

$\xi$ stands for any of the variable $S, E, I_1, I_2, I_3$, *R*. The iterative scheme for sub-systems (14) and (15) can be described as follows:

18 $$\begin{aligned} \left. \begin{array}{ll} \\ S^{n+\frac{1}{2}}_{j} &{}=S^{n}_{j}+d\tilde{t}\left( \Pi -\lambda ^{n}_{j} S^{n}_{j} -dS^{n}_{j}\right) ,\\ \\ E^{n+\frac{1}{2}}_{j} &{}=E^{n}_{j}+d\tilde{t}\left( \lambda ^{n}_{j} S^{n}_{j} -l_1E^{n}_{j}\right) ,\\ \\ {I_1}^{n+\frac{1}{2}}_{j} &{}={I_1}^{n}_{j}+d\tilde{t}\left( r k_1 E^{n}_{j} -l_{2}{I_1}^{n}_{j}\right) ,\\ \\ {I_2}^{n+\frac{1}{2}}_{j} &{}={I_2}^{n}_{j}+d\tilde{t}\left( r k_2 E^{n}_{j} -l_3{I_2}^{n}_{j}\right) ,\\ \\ {I_3}^{n+\frac{1}{2}}_{j} &{}={I_3}^{n}_{j}+d\tilde{t}\left( r(1-k_1 -k_2) E^{n}_{j}-l_4{I_3}^{n}_{j}\right) ,\\ \\ R^{n+\frac{1}{2}}_{j} &{}=R^{n}_{j}+d\tilde{t}\left( \eta _3 {I_3}^{n}_{j}+\eta _2 {I_2}^{n}_{j}+\eta _1 {I_1}^{n}_{j} -dR^{n}_{j}\right) , \end{array} \right\} \end{aligned}$$

and

19 $$\begin{aligned} \left. \begin{array}{ll} \\ S^{n+1}_{j} &{}= S^{n+\frac{1}{2}}_{j} +D_{1}\dfrac{d\tilde{t}}{d\tilde{y}^2}\left( S^{n+\frac{1}{2}}_{j-1}-2S^{n+\frac{1}{2}}_{j} +S^{n+\frac{1}{2}}_{j+1} \right) ,\\ \\ E^{n+1}_{j} &{}= E^{n+\frac{1}{2}}_{j}+D_{2}\dfrac{d\tilde{t}}{d\tilde{y}^2}\left( E^{n+\frac{1}{2}}_{j-1}-2E^{n+\frac{1}{2}}_{j} +E^{n+\frac{1}{2}}_{j+1} \right) ,\\ \\ {I_1}^{n}_{j} &{}= {I_1}^{n+\frac{1}{2}}_{j}+D_{3}\dfrac{d\tilde{t}}{d\tilde{y}^2}\left( {I_1}^{n+\frac{1}{2}}_{j-1}-2{I_1}^{n+\frac{1}{2}}_{j} +{I_1}^{n+\frac{1}{2}}_{j+1}\right) ,\\ \\ {I_2}^{n}_{j} &{}= {I_2}^{n+\frac{1}{2}}_{j}+D_{4}\dfrac{d\tilde{t}}{d\tilde{y}^2}\left( {I_2}^{n+\frac{1}{2}}_{j-1}-2{I_2}^{n+\frac{1}{2}}_{j} +{I_2}^{n+\frac{1}{2}}_{j+1}\right) ,\\ \\ {I_3}^{n}_{j} &{}= {I_3}^{n+\frac{1}{2}}_{j}+D_{5}\dfrac{d\tilde{t}}{d\tilde{y}^2}\left( {I_3}^{n+\frac{1}{2}}_{j-1}-2{I_3}^{n+\frac{1}{2}}_{j} +{I_3}^{n+\frac{1}{2}}_{j+1}\right) ,\\ \\ R^{n+1}_{j} &{}= R^{n+\frac{1}{2}}_{j} +D_{6}\dfrac{d\tilde{t}}{d\tilde{y}^2}\left( R^{n+\frac{1}{2}}_{j-1}-2R^{n+\frac{1}{2}}_{j} +R^{n+\frac{1}{2}}_{j+1} \right) . \end{array} \right\} \end{aligned}$$

### Simulation with discussions

**Figure 3:**
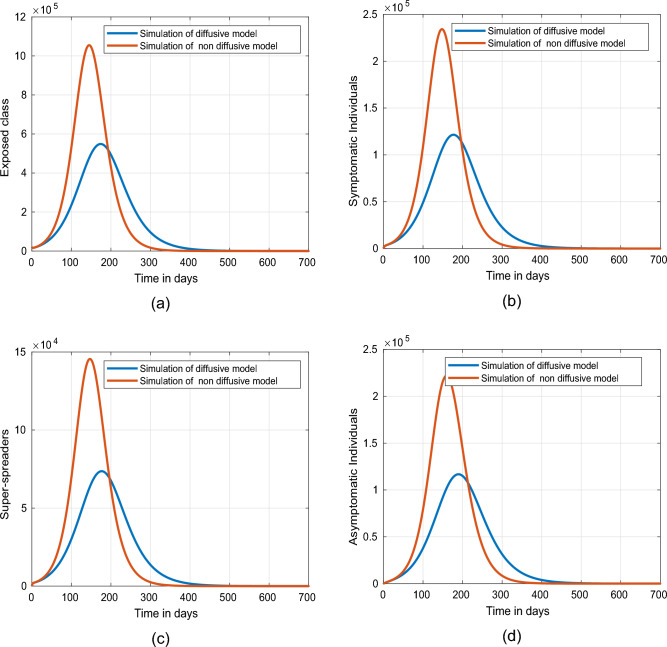
Simulation of individuals in (**a**) exposed, (**b**) symptomatic, (**c**) super-spreading, (**d**) asymptomatic sub-groups without and with diffusion.

This part presents simulation of the proposed spatio-temporal epidemic compartmental model (1) with the help of the numerical scheme discussed in (18) and (19) for the uniform and nonuniform initial conditions given by (3, 4). The impact of disease readmission rates $\beta$ and $\beta _{P}$ has been depicted for different scenarios with and without diffusion. The time resulting simulation has been conducted for super-spreaders, exposed, symptomatic and asymptomatic individuals, under the initial conditions (3, 4) with as well as without diffusion at $\tilde{y} = 0.0$ and $\tilde{y}=1.0$. The choice of considering the spatial points $\tilde{y}=0.0$ and $\tilde{y}=1.0$ is according to the initial distribution of respective populations, which indicates the region having a high density of population. Moreover, the evolutionary trajectories are obtained for 700 days. The objective is to forecast future scenarios based on confirmed infected cases and to assess the effects of the aforementioned control interventions on exposed, super-spreader, symptomatic and asymptomatic individuals without as well as with diffusion cases. Only the ICs (4) have been considered for the system with diffusion, as ICs (3) assumes a uniform population distribution. A visual dynamics of the COVID transmission model (1) is presented to explore the effectiveness of diffusion in controlling the prevalence of COVID-19 infection utilizing the initial conditions (4). The dynamics of the infected human population without and with diffusion are presented in figure 3. It can be observed from these figures that curve peaks in all cases have a significant reduction in the presence of diffusion. Physically, it reveals that public gathering restriction plays a significant impact in minimizing the infection incidence.

### Simulation for the initial conditions (3) at $\tilde{y}=0.0$ and $\tilde{y}=1.0$

**Figure 4:**
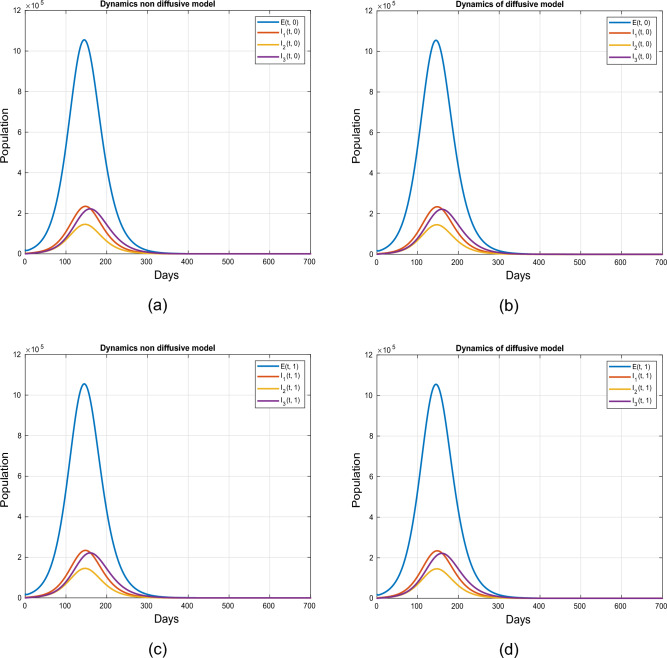
Simulation describing impact of diffusion on exposed, symptomatic, super-spreaders and asymptomatic infected individuals using initial conditions (3) at $\tilde{y}=0$ and $\tilde{y}=1$.

We provide a visual depiction of the exposed, super-spreading, symptomatic, and asymptomatic population, both without and with diffusion, using uniform ICs (3). Figure 4 demonstrate the trajectories for the model (1) for $\tilde{y}=0$ and $\tilde{y}=1$, utilizing the values outlined in the Table 2. A similar dynamics is noticed in each case as the IC (3) implies a uniform spatial distribution of the population.

### Simulation based on ICs (4) at $\tilde{y}=0.0$ with different personal protection rates

**Figure 5:**
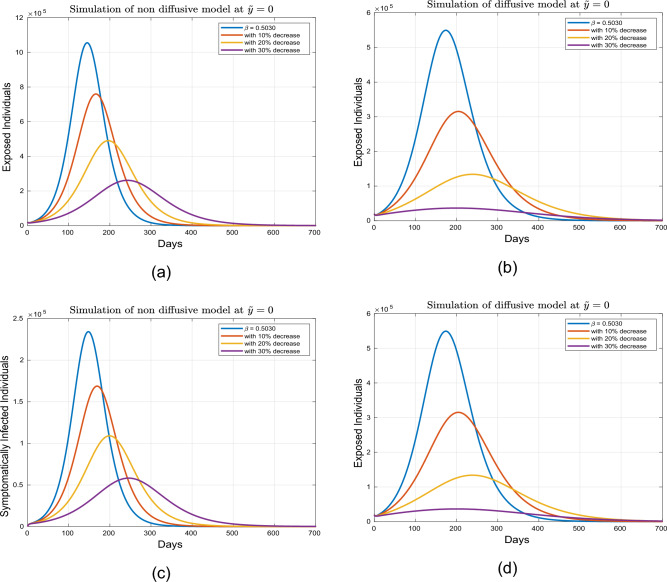
Simulation of exposed and symptomatic population under variation in $\beta$ for diffusive and non diffusive case at $\tilde{y}=0$.

**Figure 6:**
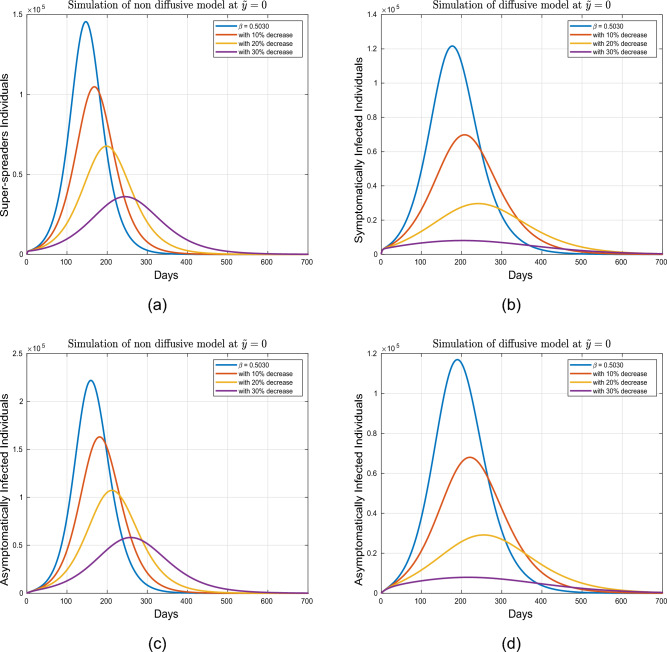
Simulation of exposed and symptomatic infected individuals under variation in $\beta$ for diffusive and non diffusive case at $\tilde{y}=0$.

**Figure 7:**
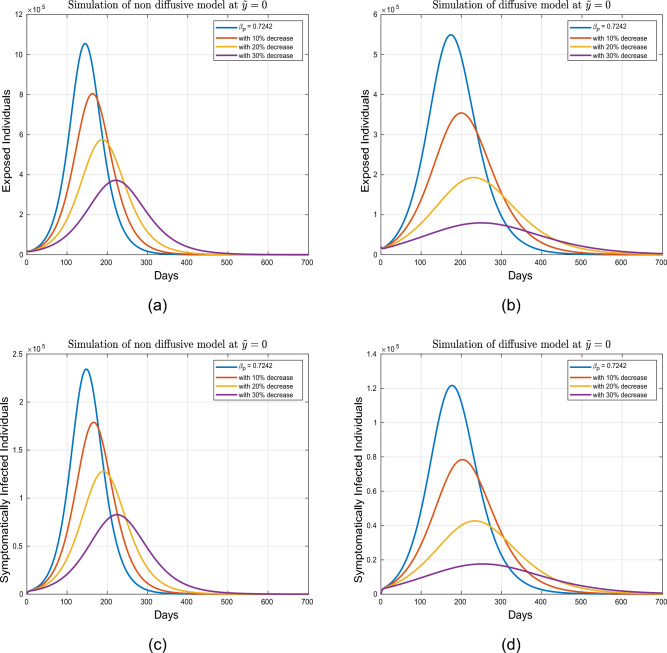
Graphical dynamics of symptomatic and exposed individuals for variation in $\beta _P$ at $\tilde{y}=0$.

**Figure 8:**
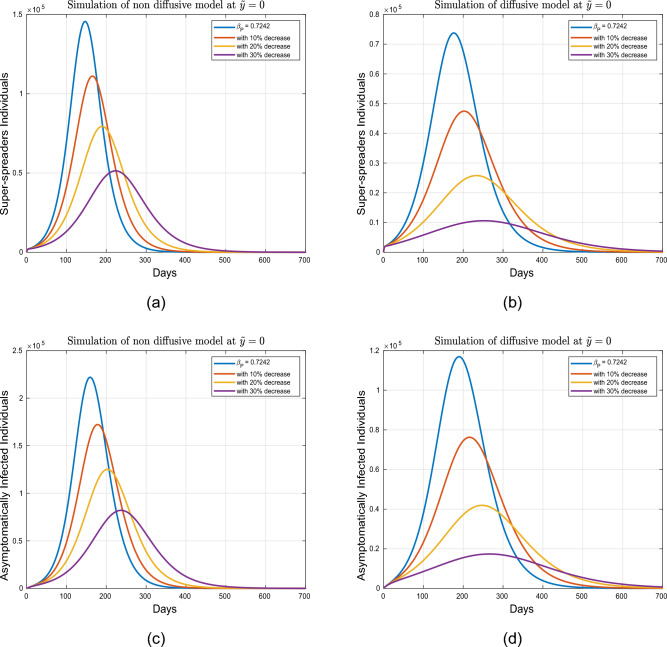
Dynamics of asymptomatic and super-spreader infected individuals under variation in $\beta _P$ for diffusive and non diffusive case at $\tilde{y}=0$.

This section accomplishes the impact of diffusion coupled with some of the model parameters for uniform and nonuniform ICs. The dynamics of the proposed model (1) for initial criterion given in (4) and under different interventions are illustrated in Figs. 5, 6, 7 and 8. These simulations are performed for both diffusive and non-diffusive scenarios at $\tilde{y}=0.0$. Figures 5 and 6 depict the influence of $\beta$ on the super-spreaders, exposed, symptomatic, and asymptomatic subgroups for both cases. Initially, the dynamics are examined for the tabulated value of $\beta$ which is set at 0.5030. Variations in $\beta$ correspond to changes in social contact intensity with increases and decreases representing relaxation and strengthening of social contacts, respectively. The impact of reducing $\beta$ by $10\%$, $20\%$, and $30\%$ is analyzed. It is observed that in the absence of diffusion, a $20\%$ reduction in $\beta$ results in a $53.01\%$ decrease in infected individuals, while a $75.0\%$ reduction is observed in the presence of diffusion. Furthermore, a $30.0\%$ reduction in social contacts leads to a $75\%$ decrease in infection without diffusion affecting exposed, super-spreaders, symptomatic, and asymptomatic infected individuals. Conversely, in the case of diffusion, a $93.01\%$ reduction is calculated with a $30.0\%$ decline in social contacts. Further analysis are presented in Tables 4 and 5. Consequently, from this analysis, it is evident that implementing isolation strategies in the presence of diffusion proves to be more advantageous and considerably contributes to mitigating the incidence of infection.

**Table 4:** Projected outcomes of individuals in the respective infected classes of model (1) in non-defeasive case.

Symbol	*E*	$I_1$	$I_2$	$I_3$	$\%$ Change to the Baseline
$\beta$ (Tabulated value)	1,055,100	234,260	145,570	221,930	–
$10\% \ \hbox {reduction in} \ \beta$	759,570	168,810	104,840	163,020	28$\%$
$20\% \ \hbox {reduction in} \ \beta$	490,380	109,080	67,712	107,260	54$\%$
$30\% \ \hbox {reduction in} \ \beta$	261,390	58,180	36,103	58,129	75$\%$
$\beta _{P}$ (baseline value)	1,055,100	234,260	145,570	221,930	–
$10\% \ \hbox {reduction in} \ \beta _P$	804,920	178,870	111,100	172,280	23$\%$
$20\% \ \hbox {reduction in} \ \beta _P$	575,060	127,880	79,396	125,110	45$\%$
$30\% \ \hbox {reduction in} \ \beta _P$	371,720	82,714	51,336	82,063	64$\%$

Figures 7 and 8 present the impact of $\beta _P$ upon the population dynamics spatially at $\tilde{y}=0.0$. Firstly, the visual dynamics are illustrated for the value of $\beta _P$ mentioned in Table 2 in diffusive as well as in non-diffusive models. The dynamics further analyzed for $10\%, 20\%$ and $30\%$ reduction in $\beta _{P}$. The simulation indicates that without diffusion case, the infected population in each compartment experiences reductions of $23\%$, $45\%$, and $64\%$, respectively. However, in the presence of diffusion, reductions of $35\%$, $64\%$, and $85\%$ are observed in the corresponding compartments. The projected numbers are summarized in Tables 4 and 5.

**Table 5:** Projected outcomes of individuals in the respective infected classes of model (1) in defeasive case.

Symbol	*E*	$I_3$	$I_2$	$I_1$	$\%$ Variation to the Baseline
$\beta$ (Table value)	548,700	73,651	116,770	121,490	–
$10\% \ \hbox {decrease in} \ \beta$	314,610	42,150	67,899	69,675	42.1$\%$
$20\% \ \hbox {decrease in} \ \beta$	133,480	17,836	29,089	29,560	75.01$\%$
$30\% \ \hbox {decrease in} \ \beta$	36,359	4842	7948	8048	93.01$\%$
$\beta _{P}$ (baseline value)	548,700	121,490	116,770	73,651	–
$10\% \ \hbox {decrease in} \ \beta _P$	353,500	47,373	76,126	78,285	35.1$\%$
$20\% \ \hbox {decrease in} \ \beta _P$	192,400	25,727	41,804	42,607	64.01$\%$
$30\% \ \hbox {decrease in} \ \beta _P$	79,245	10,567	17,303	17,545	85.00$\%$

### Visual dynamics for initial condition (4) at $\tilde{y}=1.0$ with different personal protection rates

**Figure 9:**
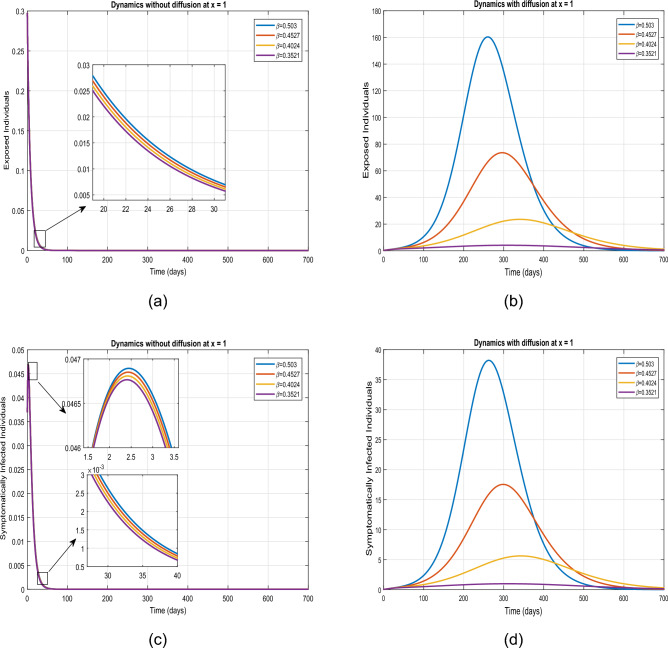
Impact of parameter $\beta$ over the solutions of exposed and symptomatic population with and without diffusion and $\tilde{y}=1$.

**Figure 10:**
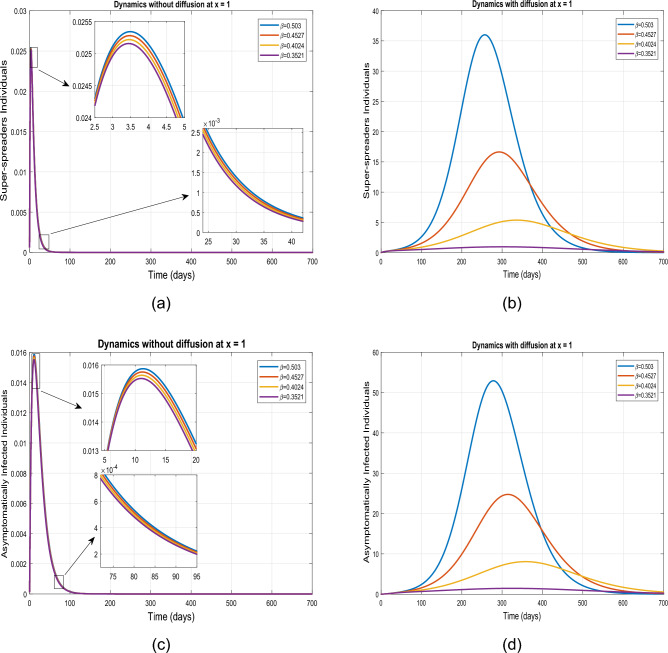
Role of $\beta$ on asymptomatic and super-spreader individuals dynamics with and without diffusion at $\tilde{y}=1$.

**Figure 11:**
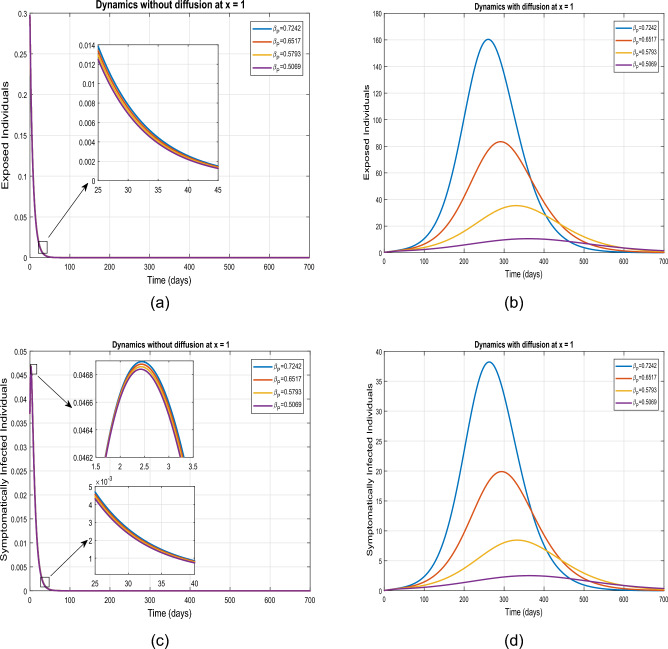
Impact of parameter $\beta _P$ on the dynamical behavior of exposed and symptomatic population with and without diffusion at $\tilde{y}=1$.

**Figure 12:**
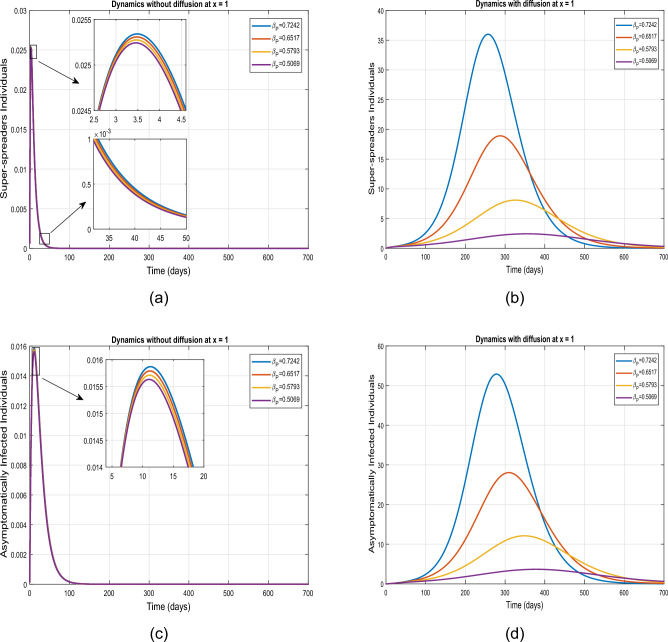
Impact of parameter $\beta _P$ on the dynamical behavior of asymptomatic and super-spreading individuals without and with diffusion at $\tilde{y}=1$.

This part of the paper presents the dynamical aspects of individuals in the aforementioned population groups at $\tilde{y}=1.0$ for 700 days. These results are graphically depicted in Figs. 9, 10, 11 and 12. Figures 9 and 10 describe the behavior of individuals in exposed, super-spreaders symptomatic and asymptomatic classes which are presented initially for $\beta =0.5030$ without and with diffusion. To further assess the role on the respective infected classes, reductions of $10\%, 20\%$, and $30\%$ are implemented. In the absence of diffusion, as depicted by the initial concentration profile in Fig. 2, at $\tilde{y}=1.0$, the low population concentration in compartments $E, I_1, I_2$, and $I_3$ results in a significant decline in the number of infected individuals with a $30\%$ decrease in the effective transmission rate $\beta$. Conversely, when the population undergoes diffusion, the super-spreader, exposed, symptomatic and asymptomatic infectious individuals observed an increase for $\beta =0.5030$. However, the number of people in these classes effectively declined with a $30\%$ decrease in $\beta$. Therefore, it can be concluded that implementing moderate social distancing policies is beneficial in reducing the number of infections in either scenario. The projected numbers for this case are presented in Tables 6 and 7.

**Table 6:** Projected peaks of the respective population classes of the model (1) at $\tilde{y}=1.0$ and without diffusion.

Symbol	*E*	$I_1$	$I_2$	$I_3$	$\%$ Variation to baseline value
$\beta$ (tabulated value)	0.2975	0.0469	0.0253	0.0159	–
$10.0\% \ \hbox {decrease }$	0.2977	0.0467	0.0253	0.0158	0.60 $\%$
$20.0\% \ \hbox {decrease }$	0.2975	0.0467	0.0252	0.0156	1.80 $\%$
$30.0\% \ \hbox {decrease }$	0.2974	0.0466	0.0251	0.0155	2.50 $\%$
$\beta _{P}$ (tabulated value)	0.2976	0.0469	0.0253	0.0159	–
$10\% \ \hbox {decrease }$	0.2977	0.0468	0.0253	0.0158	0.60 $\%$
$20\% \ \hbox {decrease }$	0.2975	0.0467	0.0252	0.0157	1.80 $\%$
$30\% \ \hbox {decrease }$	0.2974	0.0466	0.0252	0.0157	2.50 $\%$

**Table 7:** Projected peaks of the respective population classes of the model (1) at $\tilde{y}=1.0$ with diffusion.

Symbol	*E*	$I_1$	$I_2$	$I_3$	$\%$ variation to Baseline value
$\beta$ (tabulated value)	160.392	38.211	35.998	52.902	–
$10\% \ \hbox {decrease }$	73.558	17.538	16.633	24.734	54.0 $\%$
$20\% \ \hbox {decrease }$	23.563	5.622	5.366	8.064	85.0 $\%$
$30\% \ \hbox {decrease }$	4.131	0.987	0.9573	1.446	97.0 $\%$
$\beta _{P}$ (tabulated value)	160.392	38.211	35.998	52.902	–
$10\% \ \hbox {decrease }$	83.447	19.902	18.914	28.060	47.0 $\%$
$20\% \ \hbox {decrease }$	35.396	8.451	8.102	12.122	77.0 $\%$
$30\% \ \hbox {decrease }$	10.534	2.518	2.440	3.671	93.0 $\%$

Figures 11 and 12 visualize the influence of the infection transmission rate $\beta _P$ upon the dynamics super-spreaders, exposed, symptomatic, and asymptomatic infected individuals. The simulation are performed for varying $\beta _{P}$, with a reduced by $10.0\%, 20.0\%$, and $30.0\%$ relative to the tabulated value. In case of no diffusion, the super-spreaders, exposed, symptomatic, and asymptomatic infected individuals experience a clear decrease due to the lower concentration level at $\tilde{y}=1.0$, as depicted by the initial concentration profile in plot 2. Conversely, with the diffusion, the infectious individuals are increased at $\tilde{y}=1.0$ for $\beta _{P}=0.7242$. With reductions in the aforementioned parameter, a decrease in infected individuals is observed in the respective compartments, with the lowest peak observed with a $30\%$ decrease in $\beta _{P}$.

**Figure 13:**
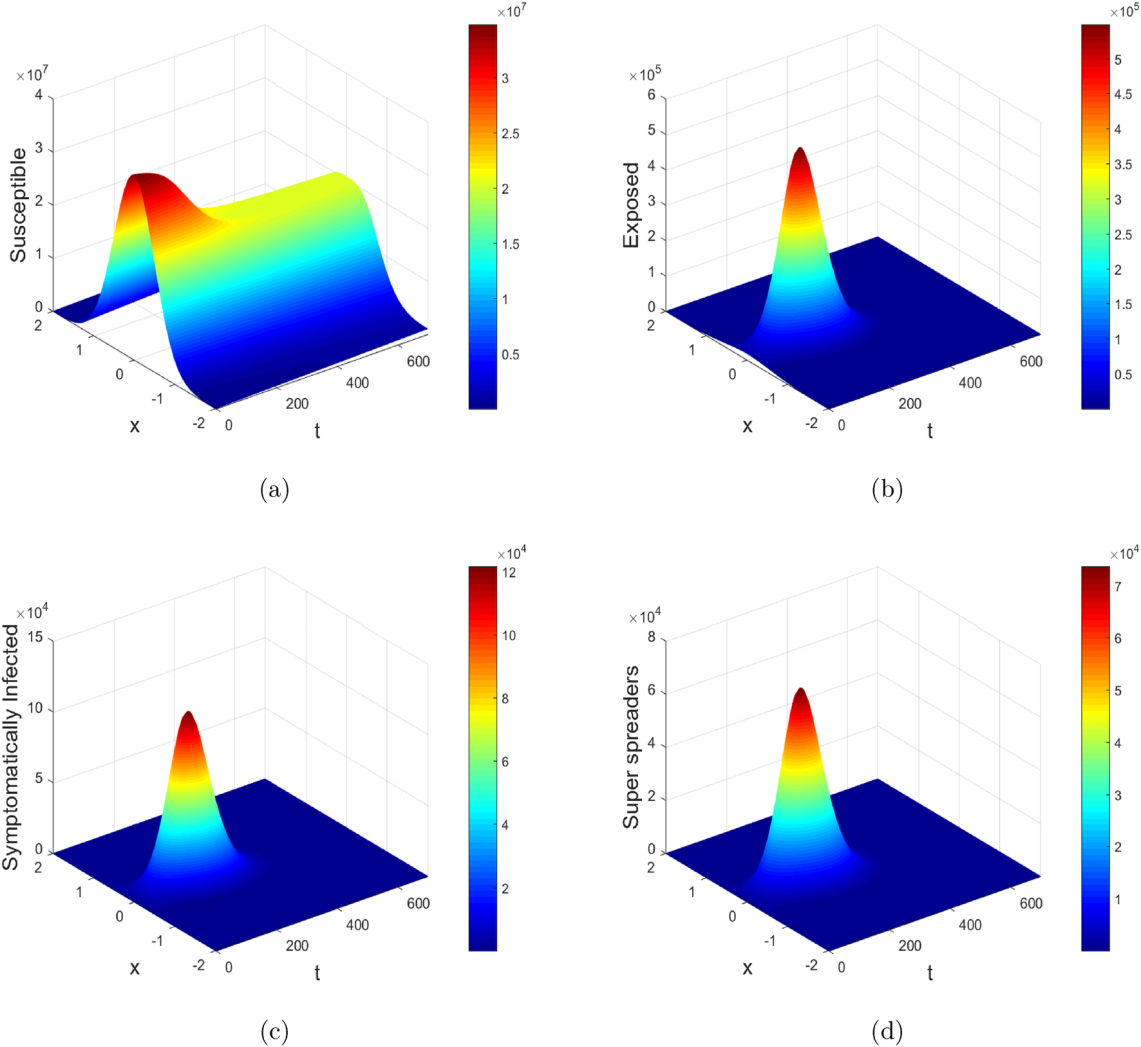
Mesh plots of susceptible, exposed, symptomatic and super-spreaders population in the presence of diffusion.

**Figure 14:**
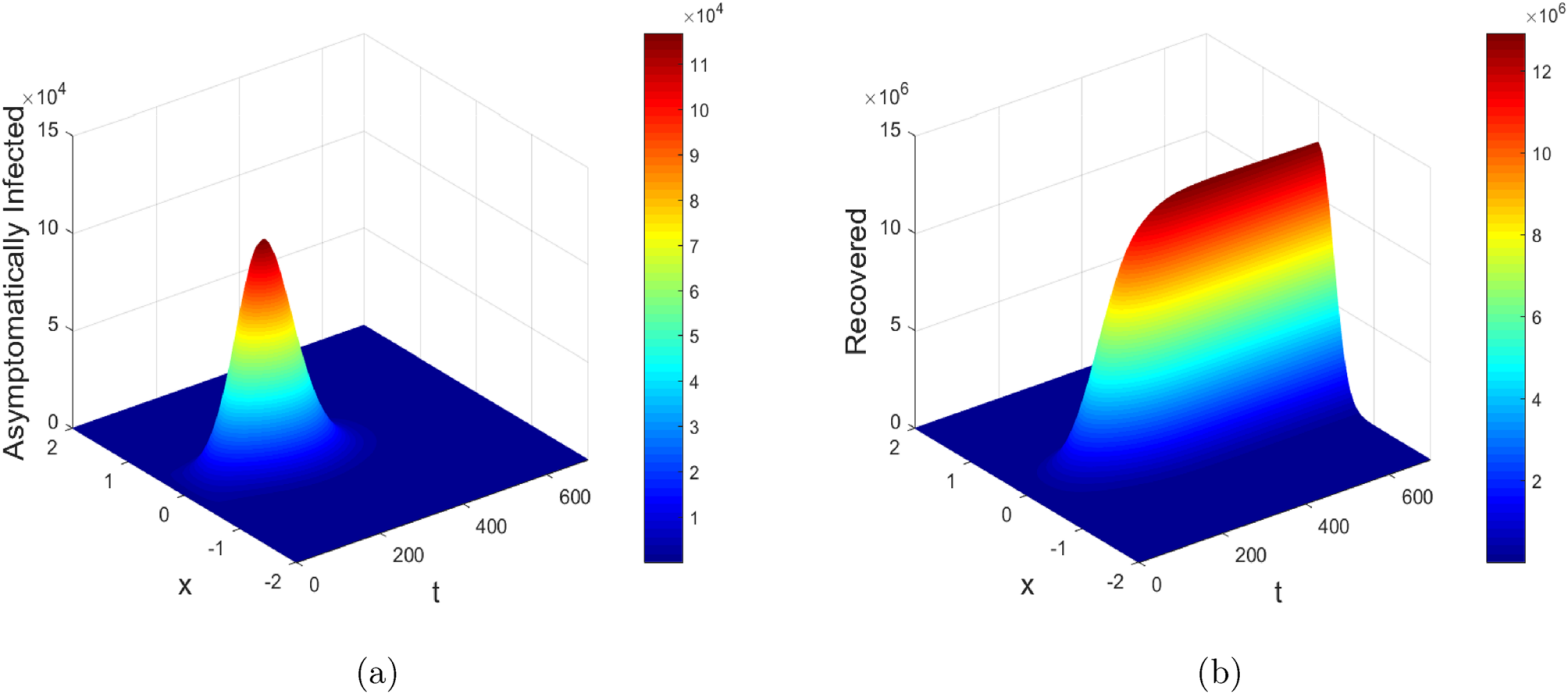
Mesh plots of asymptomatically infected and recovered individuals with diffusion.

Figures 13 and 14 show the mesh plots of the reaction-diffusion model (1). The corresponding plots indicate the spatio-temporal evolution of the proposed model over the domain $[a, b] \times [0, T_{\text {max}}]$, where $a = -2$, $b = 2$ and $T_{\text {max}} = 700$ days are taken for simulation purpose in order to investigate the long term behavior of the disease. The corresponding plots agreed with the theoretical results, i.e., the solution stays positive and converges to the steady states throughout the domain. Further, the proposed numerical schemes preserve the positivity property. Moreover, the susceptible concentration is high at $\tilde{y} = 0.0$, according to initial profiles as given in Fig. 3. Therefore with baseline values of $\beta$ and $\beta _P$, the number of infected individuals in the respective compartments gets reduced with diffusion at $\tilde{y} = 0.0$ and almost vanishes in the first 200 days. Thus diffusion will possibly curtail the infection in highly populated areas as it restricts public gatherings.

## Conclusion

The proposed study is focused on the analysis of the dynamics of COVID-19 in a spatially heterogeneous case. The impact of some non-pharmaceutical interventions (such as personal protection, isolation, etc.) is observed with and without spatial effects. For this purpose, a spatio-temporal epidemic model is formulated, consisting of a system of partial differential equations describing the dynamics of populations with different disease statuses, presented by (1). The model (1) is examined qualitatively and numerically. The main finding of the conducted study is listed as:

- The basic reproductive number $\mathfrak {R}^0$ is derived and estimate its approximate numerical value 1.3 by using values of parameters provided in Table 2. Moreover, the stability of steady-states of the proposed model (1) is discussed. It has been proved that a disease-free state is locally asymptomatically stable if $\mathfrak {R}^0<1$ and an endemic state is stable if $\mathfrak {R}^0>1$
- Sensitivity analysis of the basic reproductive number $\mathfrak {R}^0$ versus model parameters is carried out. The most sensitive parameters found are $\beta$ (disease transmission due to symptomatically and asymptomatically infected) and $\beta _{P}$ (disease transmission due to super-spreaders). It is concluded that reducing these effective contacts by isolating the infected individuals will help in reducing the transmission of infection.
- The dynamics of infected individuals are obtained spatially at $\tilde{y}=0$, and $\tilde{y}=1$ under different interventions scenarios. Furthermore, from the dynamics of respective classes, the effects of control measures $\beta$ and $\beta _{P}$ are observed in both with and with diffusion cases. It is noticed that implementing these suggested control strategies with diffusion is more effective as compared without diffusion. i.e. the number of infected individuals reduces quickly in respective infected compartments. Thus it is concluded that diffusion of population in parallel with social distancing policy plays an important role in controlling and eradicating COVID-19 infection.

The present work can be extended to fractional diffusion problems using various operators for better understanding and control of the pandemic.

## Data Availability

The data that support the findings of this study are available from the corresponding author upon reasonable request. Further, no experiments on humans and/or the use of human tissue samples involved in this study.

## References

[1] World Health Organization (WHO). https://www.who.int/emergencies/diseases/novel-coronavirus-2019/technical-guidance2020 (2020).

[2] Lin J, Yan K, Zhang J, Cai T, Zheng J (2020). A super-spreader of covid-19 in ningbo city in china. J. Infect. Public Health.

[3] Stein RA (2011). Super-spreaders in infectious diseases. Int. J. Infect. Dis..

[4] Mkhatshwa, T. & Mummert, A. Modeling super-spreading events for infectious diseases: Case study sars. arXiv:1007.0908 (2023).

[5] Zafar ZUA, Yusuf A, Musa SS, Qureshi S, Alshomrani AS, Baleanu D (2023). Impact of public health awareness programs on COVID-19 dynamics: A fractional modeling approach. FRACTALS (fractals).

[6] Zafar ZUA, DarAssi MH, Ahmad I, Assiri TA, Meetei MZ, Khan MA, Hassan AM (2023). Numerical simulation and analysis of the stochastic hiv/aids model in fractional order. Results Phys..

[7] Baba IA, Nasidi BA (2021). Fractional order epidemic model for the dynamics of novel COVID-19. Alex. Eng. J..

[8] Ibrahim A, Humphries UW, Ngiamsunthorn PS, Baba IA, Qureshi S, Khan A (2023). Modeling the dynamics of COVID-19 with real data from Thailand. Sci. Rep..

[9] Wang W, Cai Y, Wu M, Wang K, Li Z (2012). Complex dynamics of a reaction-diffusion epidemic model. Nonlinear Anal. Real World Appl..

[10] Majid F, Deshpande AM, Ramakrishnan S, Ehrlich S, Kumar M (2021). Analysis of epidemic spread dynamics using a pde model and COVID-19 data from Hamilton county OH USA. Ifac-papersonline.

[11] Zafar ZUA, Zaib S, Hussain MT, Tunç C, Javeed S (2022). Analysis and numerical simulation of tuberculosis model using different fractional derivatives. Chaos Solit. Fract..

[12] Wang N, Zhang L, Teng Z (2021). Dynamics in a reaction-diffusion epidemic model via environmental driven infection in heterogenous space. J. Biol. Dyn..

[13] Fitzgibbon W, Morgan J, Webb G, Wu Y (2020). Analysis of a reaction-diffusion epidemic model with asymptomatic transmission. J. Biol. Syst..

[14] Zheng T, Luo Y, Zhou X, Zhang L, Teng Z (2023). Spatial dynamic analysis for COVID-19 epidemic model with diffusion and beddington-deangelis type incidence. Commun. Pure Appl. Anal..

[15] Kevrekidis PG, Cuevas-Maraver J, Drossinos Y, Rapti Z, Kevrekidis GA (2021). Reaction-diffusion spatial modeling of COVID-19: Greece and andalusia as case examples. Phys. Rev. E.

[16] Baba IA, Rihan FA (2022). A fractional-order model with different strains of COVID-19. Phys. A.

[17] Ahmed N, Tahira S, Rafiq M, Rehman M, Ali M, Ahmad M (2019). Positivity preserving operator splitting nonstandard finite difference methods for seir reaction diffusion model. Open Math..

[18] Ahmed N, Fatima M, Baleanu D, Nisar KS, Khan I, Rafiq M, Ahmad MO (2020). Numerical analysis of the susceptible exposed infected quarantined and vaccinated (seiqv) reaction-diffusion epidemic model. Front. Phys..

[19] Ahmed N, Elsonbaty A, Raza A, Rafiq M, Adel W (2021). Numerical simulation and stability analysis of a novel reaction-diffusion COVID-19 model. Nonlinear Dyn..

[20] Samsuzzoha M, Singh M, Lucy D (2012). A numerical study on an influenza epidemic model with vaccination and diffusion. Appl. Math. Comput..

[21] Nawaz Y, Arif MS, Abodayeh K, Shatanawi W (2021). An explicit unconditionally stable scheme: Application to diffusive COVID-19 epidemic model. Adv. Differ. Equ..

[22] Alshehri A, Ullah S (2023). A numerical study of COVID-19 epidemic model with vaccination and diffusion. Math. Biosci. Eng..

[23] Ahmed I, Baba IA, Yusuf A, Kumam P, Kumam W (2020). Analysis of caputo fractional-order model for COVID-19 with lockdown. Adv. Differ. Equ..

[24] Zarin R, Humphries UW (2023). A robust study of dual variants of sars-cov-2 using a reaction-diffusion mathematical model with real data from the USA. Eur. Phys. J. Plus.

[25] Khan AA, Ullah S, Altanji M, Amin R, Haider N, Alshehri A, Riaz MB (2023). A numerical study of spatio-temporal COVID-19 vaccine model via finite-difference operator-splitting and meshless techniques. Sci. Rep..

[26] Li X-P, Ullah S, Zahir H, Alshehri A, Riaz MB, Al Alwan B (2022). Modeling the dynamics of coronavirus with super-spreader class: A fractal-fractional approach. Results Phys..

[27] Haider N (2014). Numerical solution of compartmental models by meshless and finite difference methods. Appl. Math. Comput..

[28] Huang W, Han M, Liu K (2009). Dynamics of an sis reaction-diffusion epidemic model for disease transmission. Math. Biosci. Eng..

[29] Groeger J (2014). Divergence theorems and the supersphere. J. Geom. Phys..

[30] Zhang L, Xing Y (2020). Stability analysis of a reaction-diffusion heroin epidemic model. Complexity.

